# VEMcomp: a Virtual Elements MATLAB package for bulk-surface PDEs in 2D and 3D

**DOI:** 10.1007/s11075-024-01919-4

**Published:** 2024-08-31

**Authors:** Massimo Frittelli, Anotida Madzvamuse, Ivonne Sgura

**Affiliations:** 1 Department of Mathematics and Physics “E. De Giorgi”, Via per Arnesano, University of Salento, 73100 Lecce, Italy; 2 Mathematics Department, The University of British Columbia, Vancouver, British Columbia Canada; 3 Department of Mathematics and Applied Mathematics, University of Pretoria, Pretoria, 0132 South Africa; 4 Department of Mathematics and Applied Mathematics, University of Johannesburg, PO Box 524, Auckland Park, 2006 South Africa; 5 Department of Mathematics and Computational Science, Faculty of Science, University of Zimbabwe, Mount Pleasant, Harare, Zimbabwe

**Keywords:** Virtual element method, Bulk-surface virtual element method, Bulk-surface finite element method, Bulk-surface PDEs, Mesh generation, IMEX Euler Method, MATLAB

## Abstract

We present a Virtual Element MATLAB solver for elliptic and parabolic, linear and semilinear Partial Differential Equations (PDEs) in two and three space dimensions, which is coined VEMcomp. Such PDEs are widely applicable to describing problems in material sciences, engineering, cellular and developmental biology, among many other applications. The library covers linear and nonlinear models posed on different simple and complex geometries, involving time-dependent bulk, surface, and bulk-surface PDEs. The solver employs the Virtual Element Method (VEM) of lowest polynomial order ${k=1}$ on general polygonal and polyhedral meshes, including the Finite Element Method (FEM) of order ${k=1}$ as a special case when the considered mesh is simplicial. VEMcomp has three main purposes. First, VEMcomp generates polygonal and polyhedral meshes optimized for fast matrix assembly. Triangular and tetrahedral meshes are encompassed as special cases. For surface PDEs, VEMcomp is compatible with the well-known Matlab package DistMesh for mesh generation. Second, given a mesh for the considered geometry, possibly generated with an external package, VEMcomp computes all the matrices (mass and stiffness) required by the VEM or FEM method. Third, for multiple classes of stationary and time-dependent bulk, surface and bulk-surface PDEs, VEMcomp solves the considered PDE problem with the VEM or FEM in space and IMEX Euler in time, through a user-friendly interface. As an optional post-processing, VEMcomp comes with its own functions for plotting the numerical solutions and evaluating the error when possible. An extensive set of examples illustrates the usage of the library.

## Introduction

The Virtual Element Method (VEM) was first proposed in [1] for elliptic problems in two space dimensions as a generalization of the Finite Element Method (FEM), where the mesh elements can be general polygons instead of triangles. The usage of polygons with arbitrary number of edges is made possible by enriching the local space of polynomials with suitable non-polynomial functions defined as solutions of an element-wise problem. This elegant idea ensures the optimal polynomial accuracy of the method. The immediate success of VEM is due to the multiple benefits of its geometric flexibility. Among such benefits, we mention: (i) efficient mesh refinement techniques [2, 3], (ii) numerical solutions with high global regularity [4–6], (iii) accurate approximation of boundaries [7–11], (iv) easy mesh pasting [12, 13], and (v) easy handling of complex domain shapes and cuts [10, 14].

Motivated by its multiple advantages, the VEM was quickly extended to numerous Partial Differential Equations (PDE) problems and applications. A non-exhaustive list of models for which the VEM has been employed comprises of (i) linear elliptic problems in two [1, 7] and three [15] space dimensions, (ii) semilinear elliptic problems in two and three space dimensions [16], (iii) linear heat equation in two [17] and three [18] space dimensions, (iv) semilinear parabolic equations [19] and reaction-diffusion systems [20], (v) elasticity [21, 22] and plasticity [23] problems, (vi) phase-field models [6, 24, 25], (vii) fluid dynamics [26, 27], (viii) fracture models [14], (ix) surface [13, 28] and bulk-surface [29–32] PDEs, and recently (x) PDEs on evolving flat domains [33].

**Fig. 1:**
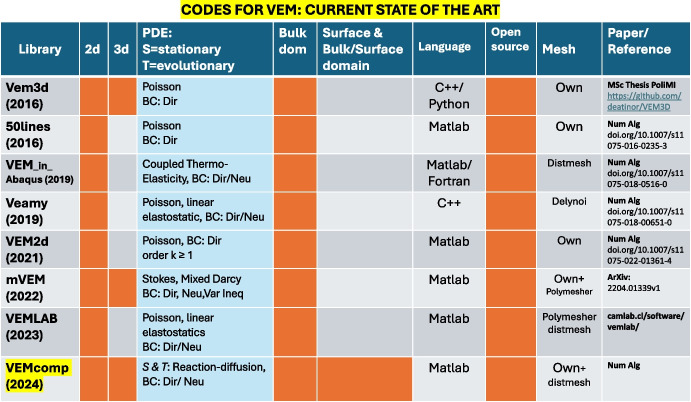
State of the art of the existing VEM solvers

Over the last ten years, VEM has established itself as a reliable numerical method with desirable properties for solving PDEs. This has stimulated the development of the first open-source VEM libraries and codes. Here we will recall some of the current state-of-the-art libraries and then outline the key contributions of VEMcomp to existing libraries as well as its major differences and improvements. The work in [34] presents a MATLAB implementation of the baseline VEM application: the lowest order VEM for the Poisson problem in 2D. For the same problem, a high order VEM code in MATLAB is then provided in [35]. An Abaqus-MATLAB VEM code for coupled thermo-elasticity problems in 2D is presented in [36]. VEM libraries for elasticity problems in 2D are available in MATLAB/Octave [37] and C++ [38]. For PDE problems in three space dimensions (3D), we mention the MATLAB library mVEM [39] for the Poisson, Stokes equations, linear elasticity and friction problems, and the C++/Python library VEM3D [40] for the Laplace equation. All the mentioned libraries are dedicated to specific cases or applications and are confined to stationary bulk-only problems. The state of the art of VEM libraries is schematically reviewed in Fig. 1.

To the best of the authors’ knowledge, there is no open-source VEM library so far for time-dependent PDE problems in two nor three space dimensions nor for surface or bulk-surface PDE problems in 2D or 3D. In this work, we contribute to the fields of VEM and FEM open-source libraries. We propose a MATLAB library, which we call VEMcomp, for linear elliptic and linear and nonlinear parabolic PDE problems in 2D and 3D, including bulk, surface and bulk-surface PDE models. VEMcomp has four purposes:

1. **Mesh generation**: For 2D and 3D domains represented as level sets, the library generates polytopal (i.e. polygonal in 2D and polyhedral in 3D) meshes specifically optimized for fast matrix assembly, following [29, 30]. Regardless of the space dimension, the surface mesh is always taken as the boundary of the bulk mesh.
2. **Matrix assembly**: Given any polygonal or polyhedral mesh – not necessarily generated with VEMcomp itself –, the library generates all the matrices involved in the VEM and FEM methods (e.g. mass, stiffness). For example, for the case of triangulated surfaces in $\mathbb {R}^3$ with an empty boundary, VEMcomp is fully compatible with the triangulated surfaces generated by the well-known Matlab package DistMesh [41];
3. **Black-box solvers**: For multiple classes of PDE problems, the library provides a black-box interface that allows the user to set the problem parameters, and returns the VEM or FEM numerical solution. For time-dependent problems, the time discretization is carried out with the IMplicit-EXplicit (IMEX) Euler method, which has been proven to be simple and effective in combination with FEMs and VEMs for surface [42, 43] and bulk-surface PDEs [29, 30];
4. **Post-processing**: The library can, through inhouse functions, plot the numerical solution and compute the relative error in $L^2$ norm if the exact solution is known in closed form, without having to resort to external software.

**Fig. 2:**
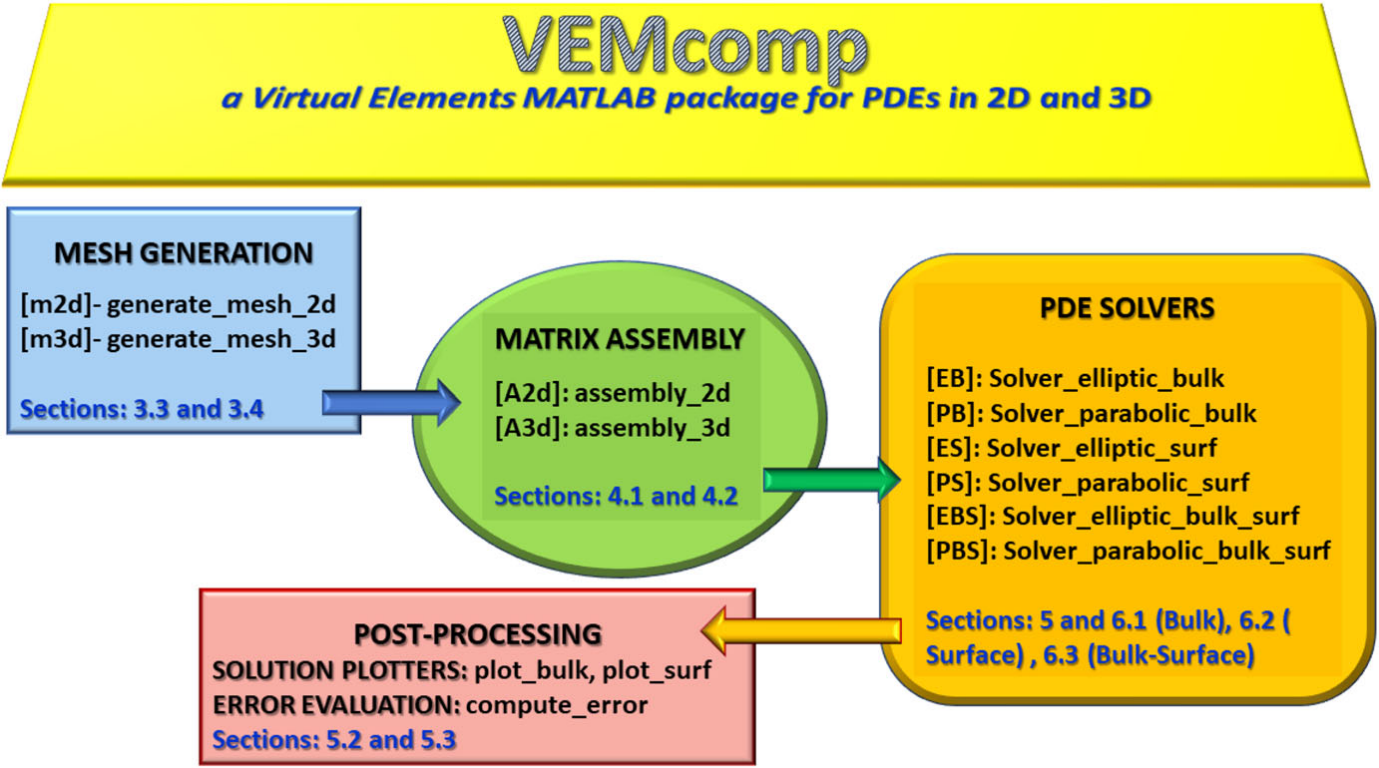
Schematic representation of VEMcomp’s three facilities: (i) polytopal mesh generation, (ii) matrix assembly and (iii) built-in solvers. Each of these facilities is composed of functions that execute specific tasks, as shown in the picture

For the sake of clarity, a sketch of the VEMcomp structure together with references to the corresponding sections in the main text of the paper, is reported in Fig. 2.

The structure of our paper is as follows. In Section 2, we state the multiple model problems to be addressed in this work, thereby motivating the functionalities of VEMcomp. In Section 3, we illustrate how VEMcomp generates polytopal (i.e. polygonal in 2D and polyhedral in 3D) meshes, including simplicial (i.e. triangular in 2D and tetrahedral in 3D) meshes. Section 4 showcases VEMcomp’s ability to compute local and global VEM and FEM matrices. In Section 5, we present VEMcomp’s user-friendly solvers, solution plotters, and error evaluation functions for the model problems outlined in Section 2. Section 6 lists several numerical examples that illustrate at once the usage of VEMcomp. In Section 7, we draw our conclusions and outline future research directions.

## Overview

VEMcomp is an object-oriented VEM library written in MATLAB. Compared to other existing VEM libraries, VEMcomp aims to fill the gap for PDE problems (i) with time-dependence and (ii) on complex geometries, where surface or bulk-surface PDEs are posed. In the remainder of this section, let $\Omega \subset \mathbb {R}^d$, $d=2,3$, be a compact domain in the *d*-dimensional Euclidean space. The first class of PDE problems covered by VEMcomp comprises linear elliptic bulk-only PDEs, and is given by

1 $$\begin{aligned} {\left\{ \begin{array}{ll} - d^{\Omega }\Delta u +\alpha u = f(\varvec{x}), \quad \varvec{x}\in \Omega ;\\ u(\varvec{x}) = 0 \quad \text {or} \quad \dfrac{\partial u}{\partial \varvec{n}}(\varvec{x}) = 0, \quad \varvec{x}\in \partial \Omega ,\\ \end{array}\right. } \end{aligned}$$

where $\Delta$ denotes the Laplace operator in $\Omega$, $d^{\Omega } > 0$ is a positive diffusion coefficient, $\alpha \ge 0$ is a nonnegative coefficient, and *f* is a sufficiently smooth source term. As a time-dependent counterpart of (1), VEMcomp covers semilinear parabolic bulk-only PDE systems of $n\in \mathbb {N}$ equations of the following form

2 $$\begin{aligned} {\left\{ \begin{array}{ll} \dfrac{\partial u_{i}}{\partial t} - d_{i}^{\Omega }\Delta u_{i} = f_{i}(u_{1},\dots , u_{n}, \varvec{x}, t), \quad (\varvec{x},t) \in \Omega \times [0,T], \\ u_{i}(\varvec{x}, t) = 0 \quad \text {or} \quad \dfrac{\partial u_{i}}{\partial \varvec{n}}(\varvec{x}, t) = 0, \quad (\varvec{x},t) \in \partial \Omega \times [0,T],\\ u_{i}(\varvec{x}, 0) = u_{i,0}(\varvec{x}), \qquad \varvec{x}\in \Omega , \end{array}\right. } \end{aligned}$$

for $i=1,\dots ,n$, where $d_{1}^{\Omega },\dots , d_{n}^\Omega > 0$ are diffusion coefficients, $T>0$ is the final time, $f_{1}, \dots , f_{n}$ are smooth enough linear or nonlinear functions, and $u_{1,0}, \dots , u_{n,0}$ are sufficiently smooth initial data. The general models (1) and (2) comprise several notable bulk-only PDE problems that were solved with VEM in the literature, such as (i) linear [1, 7, 15] and semilinear elliptic problems [16], (ii) linear [17] and semilinear parabolic problems [19] including reaction-diffusion systems [20].

If $\Gamma = \partial \Omega$ is a sufficiently smooth manifold with an empty boundary, a second class of PDEs that fall in VEMcomp’s framework is the following class of linear elliptic surface PDEs:

3 $$\begin{aligned} - d^{\Gamma } \Delta _{\Gamma } v +\beta v = g(\varvec{x}), \qquad \varvec{x}\in \Gamma , \end{aligned}$$

where $\Delta _{\Gamma }$ represents the Laplace-Beltrami operator on $\Gamma$, $d^{\Gamma } > 0$ is a diffusion coefficient, $\beta > 0$ is a positive coefficient, and *g* is a regular enough source term. As a time-dependent counterpart of (3), VEMcomp solves the following class of semilinear parabolic surface PDEs or systems of $m\in \mathbb {N}$ surface PDEs:

4 $$\begin{aligned} {\left\{ \begin{array}{ll} \dfrac{\partial v_{j}}{\partial t} - d_{j}^{\Gamma } \Delta _{\Gamma } v_{j} = g_{j}(v_{1},\dots ,v_{n}, \varvec{x}, t), \qquad \varvec{x}\in \Gamma , \\ v_{j}(\varvec{x}, 0) = v_{j,0}(\varvec{x}), \qquad \varvec{x}\in \Gamma , \end{array}\right. } \end{aligned}$$

for $j=1,\dots ,m$, where $\Delta _{\Gamma }$ represents the Laplace-Beltrami operator on $\Gamma$, $d_{1}^{\Gamma },\dots , d_{m}^{\Gamma } > 0$ are diffusion coefficients, $g_{1}, \dots , g_{m}$ are smooth enough linear or nonlinear functions, $T>0$ is the final time and $v_{1,0}, \dots , v_{m,0}$ are sufficiently smooth initial data. In (3) and (4), no boundary conditions are needed since, as we mentioned, $\Gamma$ has no boundary. The general models (3) and (4) encompass several surface PDE (SPDE) models of interest, such as elliptic SPDEs [13, 28] and surface reaction-diffusion systems (SRDSs) [44, 45].

The third and most complex class of PDE problems solvable by VEMcomp is given by bulk-surface PDEs. In the stationary case, we consider linear elliptic coupled bulk-surface PDEs of the following form:

5 $$\begin{aligned} {\left\{ \begin{array}{ll} - d^{\Omega } \Delta u + \alpha u = f(\varvec{x}), \qquad \varvec{x}\in \Omega ;\\ - d^{\Gamma } \Delta _{\Gamma } v + \beta v= g(\varvec{x}), \qquad \varvec{x}\in \Gamma ;\\ \dfrac{\partial u}{\partial \varvec{n}} = \gamma u + \delta v, \qquad \varvec{x}\in \Gamma ,\\ \end{array}\right. } \end{aligned}$$

where $d^{\Omega }, d^{\Gamma } > 0$ are diffusion coefficients, $\alpha ,\beta \ge 0$ are nonnegative coefficients, $\gamma ,\delta \ge 0$ are nonnegative coupling coefficients, and $f \text {and}\, g$ are regular enough source terms. As a time-dependent counterpart of (5), VEMcomp solves the following coupled bulk-surface reaction-diffusion system (BS-RDS):

6 $$\begin{aligned} {\left\{ \begin{array}{ll} \dfrac{\partial u_{i}}{\partial t} - d^{\Omega }_{i} \Delta u_{i} = f_{i}(u_{1}, \dots , u_{n}, \varvec{x},t), \qquad \varvec{x}\in \Omega ;\\ \dfrac{\partial v_{j}}{\partial t} - d^{\Gamma }_{j} \Delta _{\Gamma } v_{j} = g_{j}(v_{1},\dots ,v_{m},\varvec{x},t), \qquad \varvec{x}\in \Gamma ;\\ \dfrac{\partial u_{i}}{\partial \varvec{n}} = h_{i}(u_{1},\dots ,u_{n},v_{1},\dots ,v_{m},\varvec{x},t), \qquad \varvec{x}\in \Gamma ;\\ u_{i}(\varvec{x},0) = u_{i,0}(\varvec{x}), \qquad \varvec{x}\in \Omega ;\\ v_{j}(\varvec{x},0) = v_{j,0}(\varvec{x}), \qquad \varvec{x}\in \Gamma , \end{array}\right. } \end{aligned}$$

for $i=1,\dots ,n$ and $j=1,\dots ,m$, where $d_{1}^{\Omega }, \dots , d^{n}_{\Omega },d_{1}^{\Gamma }, \dots , d_{m}^{\Gamma } > 0$ are diffusion coefficients, $f_{1},\dots ,f_{n}$, $g_{1},\dots ,g_m$, $h_1,\dots , h_n$ are smooth enough linear or nonlinear functions, $T > 0$ is the final time and $u_{1,0}, \dots , u_{n,0}, v_{1,0}, \dots , v_{m,0}$ are sufficiently smooth initial data. We recall that the VEM was extended to bulk-surface reaction-diffusion systems (BS-RDSs) in two [29] and three [30] space dimensions, which fall within the general class (6). These results further motivate our work.

In the next Sections we will illustrate in detail the three main facilities of VEMcomp: (i) polygonal and polyhedral mesh generation, (ii) computation of local VEM matrices and matrix assembly, and (iii) black-box solvers and solution plotters for problems (1)-(6). Each of these three facilities is composed of functions that execute specific tasks. A schematic representation of VEMcomp’s facilities and their respective functions is shown in Fig. 2. VEMcomp is compatible with MATLAB R2019a and higher.

## Element representation and mesh generation

Polygonal and polyhedral mesh generation is a niche topic, and very few off-the-shelf and easy to use software packages are available. For domains in two space dimensions, we mention the software PolyMesher [46] and Delynoi [47]. For domains in three space dimensions, we mention the software Polylla [48] and the MATLAB toolbox voronoi3d [49]. To the best of the authors’ knowledge, there is no open-source software for bulk-surface mesh generation in two or three space dimensions. For 2D and 3D domains in level set form, VEMcomp fills this gap. We point out that, when restricted to triangular (in 2D) or tetrahedral meshes (in 3D), the Virtual Element Method (VEM) of low polynomial order $k=1$ boils down to the Finite Element Method (FEM). This means that VEMcomp can be used as a FEM solver for surface and bulk-surface PDEs, when provided with triangular/tetrahedral meshes, for which several open-source generators exist, for instance the Matlab package DistMesh [41] as illustrated in the numerical example in Section 6.2.2. We start by presenting basic classes that allow to represent single elements in two and three space dimensions.

### The class element2d

The class element2d represents a polygonal element in 2D. It contains minimal information that uniquely identify the element. To create an instance, say obj, of the class element2d, use one of the following constructors:
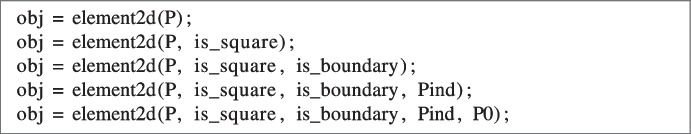
 where the inputs are defined as follows:

- P is a NVert $\times$ 3 array containing the coordinates of the vertexes, ordered clockwise or counterclockwise. The vertexes have three coordinates, because two-dimensional elements are also faces of three-dimensional elements.
- The Boolean is_square determines if obj is a square (for which the local VEM matrices are known in closed form).
- The Boolean is_boundary determines if obj is a face of a three-dimensional element lying on the boundary $\Gamma$ of the bulk domain $\Omega$. This information is necessary for the assembly of global VEM matrices;
- If the element obj is part of a mesh, and the coordinates of all the nodes are stored into an array, say PP, of size NMesh $\times$ 3, then the property Pind contains the indexes of obj.P in PP, i.e. PP(Pind,:) = obj.P. This information is crucial for matrix assembly;
- P0 is a $3\times 1$ array that contains the coordinates of a point w.r.t. which the element obj is star-shaped. VEMcomp needs this information later for the computation of local mass and stiffness matrices. However, the condition that each 2D element is star-shaped is not restrictive, as it is a basic assumption in the VEM literature, see [50].

Once an instance obj of the class element2d has been created, its properties can be accessed using the dot syntax, e.g. P = obj.P.

### The class element3d

Analogous to the class element2d, the class element3d represents a polyhedral element in 3D. It contains minimal information that uniquely identify the element. To create an instance, say obj, of the class element3d, use one of the following constructors: 
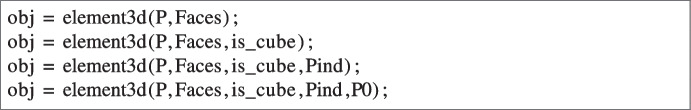
 where the inputs are defined as follows:

- P is a NVert $\times$ 3 array containing the coordinates of the vertexes in any order;
- Faces is a NFaces $\times$ 1 array of instances of the class element2d, representing the faces of obj;
- The Boolean is_cube determines if obj is a cube (for which the local VEM matrices are known in closed form).
- If the element obj is part of a mesh, and the coordinates of all the nodes are stored in an array, say PP, of size NMesh $\times$ 3, then the property Pind contains the indexes of obj.P in PP, i.e. PP(Pind,:) = obj.P;
- P0 is a $3\times 1$ array that contains the coordinates of a point w.r.t. which the element obj is star-shaped. As in the 2D case, this information is needed for the computation of local and global mass and stiffness matrices, and the assumption that each 3D element is star-shaped is common in the VEM literature, see [15].

### Mesh generation in 2D

We can now state that any polygonal mesh in 2D can be represented as a collection of element2d. Even if the user is free to write custom code to generate meshes as collections of element2d, VEMcomp comes with a built-in function for the generation of meshes in this format, for domains that are defined as level sets of Lipschitz functions as demonstrated next. Let $Q := [x_{\text {min}}, x_{\text {max}}] \times [y_{\text {min}}, y_{\text {max}}] \subset \mathbb {R}^2$ be a compact rectangle and let $f: Q \rightarrow \mathbb {R}$ be a Lipschitz function. Let $\Omega \subset \mathbb {R}^2$ and $\Gamma = \partial \Omega$ be defined respectively as

7 $$\begin{aligned} \Omega = \{\varvec{x}\in Q\ |\ f(\varvec{x}) \le 0\}, \quad \text {and}\quad \Gamma = \{\varvec{x}\in Q\ |\ f(\varvec{x}) = 0\}. \end{aligned}$$

**Fig. 3:**
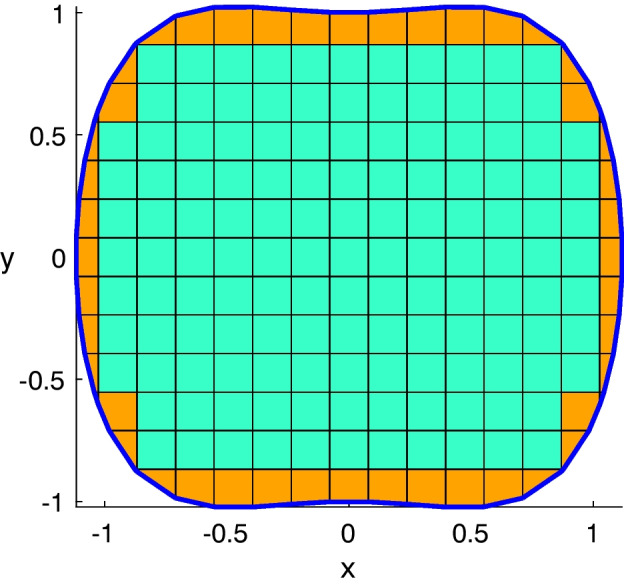
A conforming bulk-surface mesh in 2D generated with the VEMcomp function generate_mesh2d. The bulk domain $\Omega$ is approximated by a polygonal mesh $\Omega _h$ composed of square elements (green) and more general polygonal elements (orange) close to the surface $\Gamma$. The surface $\Gamma$ is approximated by a piecewise straight line $\Gamma _h$ (blue)

The rectangle *Q* is subdivided with a Cartesian grid composed of rectangular elements. Then, the rectangles that intersect the boundary $\Gamma$ are cut. This well-known algorithm, called *marching squares*, produces a piecewise linear approximation $\Gamma _h$ of the boundary $\Gamma$ [51]. However, our purpose is to produce a mesh for both the bulk $\Omega$ and the surface $\Gamma$. To this end, the rectangles and the cut rectangles produced as a by-product of the marching squares algorithm, constitute a polygonal approximation $\Omega _h$ of the bulk $\Omega$, such that the approximate boundary $\Gamma _h$ is exactly the boundary of $\Omega _h$, i.e. $\Gamma _h = \partial \Omega _h$, see Fig. 3 for an illustration. This property is typical of simplicial (i.e. triangular and tetrahedral) meshes generated with well-assessed packages like FEniCS [52] or deal.II [53]. The approach proposed here generalizes the strategy proposed in our previous work [29], which was confined to spherical domains, to general level-set domains. In fact, this new approach does not require the projection of nodes onto the surface $\Gamma$. VEMcomp generates conforming bulk-surface meshes in 2D as described above through the function generate_mesh2d, whose syntax is as follows: 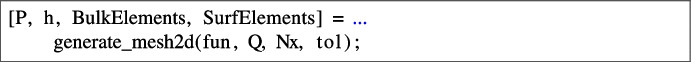

In the above, the inputs and outputs are defined as follows. In input:

- fun is the level function used in (7), represented as an inline function of the type fun = @(P) <expression>, where P is any $n\times 2$ array of $n\in \mathbb {N}$ points in $\mathbb {R}^{2}$;
- Q is the $2\times 2$ array introduced in (7), which in Matlab can be created using the syntax $Q = [x_\text {min}, x_\text {max}; y_\text {min}, y_\text {max}]$;
- Nx $>= 2$ is the required amount of gridpoints along each dimension. If *Q* is not a square, then the shortest side of *Q* is discretised with Nx gridpoints, such that the resulting grid is equally spaced;
- tol $>0$ is the minimum distance between distinct nodes. Any two nodes distant from each other less than tol will be merged into a unique node, in order to avoid elements with excessively short edges.

In output:

- P is a $N\times 2$ array containing the $N\in \mathbb {N}$ nodes of the bulk mesh $\Omega _h$. We remark that the nodes of the surface mesh $\Gamma _h$ constitute a subset of P;
- h $>0$ is the meshsize of the bulk mesh $\Omega _h$. Since the surface mesh $\Gamma _h$ is the boundary of the bulk mesh $\Omega _h$, then *h* is also an upper bound of the meshsize of the surface mesh $\Gamma _h$;
- BulkElements is a list of the bulk elements in element2d format;
- SurfElements is a $M\times 2$ array, where $M\in \mathbb {N}$ is the number of surface elements and, for each $i=1,\dots ,M$, the nodes of the *i*-th surface element (a segment) are P(SurfElements(i,:),:).

We remark that the marching squares algorithm does not guarantee shape regularity [51]. This means that shape regularity of the obtained mesh does not depend monotonically on the discretisation level $N_x$. This unpredictable behavior also depends on the shape of the domain under consideration. As a workaround, the user can try different values of the discretisation level $N_x$ and different sizes for the bounding box *Q*. To the best of our knowledge, regular mesh generation for general level-set domains is an open problem. We also remark that VEMcomp can be used in combination with an externally generated mesh, if converted to the format returned by the function generate_mesh2d described above. Finally, it must be noted that generate_mesh2d is guaranteed to generate star-shaped elements, which is essential later for matrix assembly, see Section 3.1.

### Mesh generation in 3D

In a similar way, we address mesh generation in 3D. Let $Q := [x_\text {min}, x_\text {max}] \times [y_\text {min}, y_\text {max}] \times [z_\text {min}, z_\text {max}] \subset \mathbb {R}^3$ be a compact cuboid and let $f: Q \rightarrow \mathbb {R}$ be a Lipschitz function. Let $\Omega \subset \mathbb {R}^3$ and $\Gamma = \partial \Omega$ be defined respectively as

8 $$\begin{aligned} \Omega = \{\varvec{x}\in \ Q\ |\ f(\varvec{x}) \le 0\}, \quad \text {and}\quad \Gamma = \{\varvec{x}\in Q\ |\ f(\varvec{x}) = 0\}. \end{aligned}$$

**Fig. 4:**
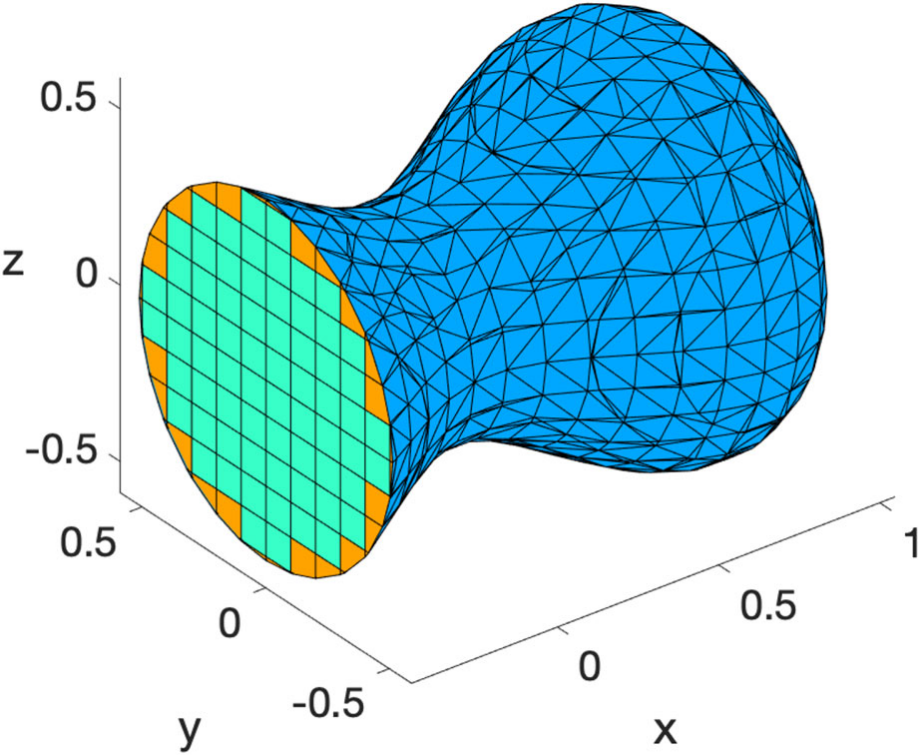
A conforming bulk-surface mesh in 3D generated with the VEMcomp function generate_mesh3d. The bulk domain $\Omega$ is approximated by a polyhedral mesh $\Omega _h$ composed of cubic elements (green) and more general polyhedral elements (orange) close to the surface $\Gamma$. The surface $\Gamma$ is approximated by a triangulated surface $\Gamma _h$ (blue) taken as the boundary of the bulk mesh $\Omega _h$


The well-known *marching cubes* algorithm [51] is often used to generate triangulated surface meshes. Similarly to the 2D case, the algorithm starts with a cubic 3D mesh and then produces a triangulated mesh $\Gamma _h$ composed of triangular faces obtained by cutting the cubic elements that intersect the surface $\Gamma$, see [51]. In our work [31] we have shown that the cubes and cut cubes produced as a by-product of the the marching cubes algorithm naturally compose a polyhedral bulk mesh $\Omega _h$ that approximates $\Omega$, such that $\Gamma _h = \partial \Omega _h$, see Fig. 4 for an illustration. This bulk-surface variant of the marching cubes algorithm was proposed in our work [31] and is now available in VEMcomp through the function generate_mesh3d, whose syntax is as follows:

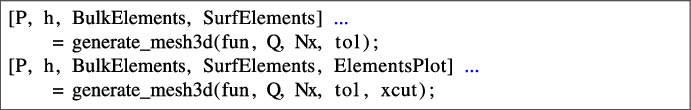


In the above, inputs and outputs are analogous to the 2D case. Specifically, in input:

- fun is the level function used in (8), represented as an inline function of the type fun = @(P) <expression>, where P is any $n\times 3$ array of $n\in \mathbb {N}$ points in $\mathbb {R}^3$;
- Q is the $2\times 3$ array introduced in (8), which in Matlab syntax can be created using the syntax $Q = [x_\text {min}, x_\text {max}; y_\text {min}, y_\text {max}; z_\text {min}, z_\text {max}]$;
- Nx $>= 2$ is the required amount of gridpoints along each dimension. If *Q* is not a cube, then the shortest side of *Q* is discretised with Nx gridpoints, such that the resulting grid is equally spaced;
- tol $>0$ is the minimum distance between distinct nodes. Any two nodes distant from each other less than tol will be merged into a unique node;
- If the optional input xcut$\in \mathbb {R}$ is provided, then VEMcomp considers the auxiliary mesh
  9 $$\begin{aligned} \Omega _\text {cut} := \{(x,y,z) \in \Omega \ | \ x \le x_\text {cut}\}, \qquad \Gamma _\text {cut} := \partial \Omega _\text {cut}, \end{aligned}$$
  which will be useful at a later stage to plot numerical solutions inside of $\Omega$, as explained below in the list of outputs.

In output:

- P is a $N\times 3$ array containing the $N\in \mathbb {N}$ nodes of the bulk mesh $\Omega _h \subset \mathbb {R}^3$. The nodes of the surface mesh $\Gamma _h$ constitute a subset of P;
- h $>0$ is the meshsize of the bulk mesh $\Omega _h$;
- BulkElements is a list of the bulk elements in element3d format;
- SurfElements is a $M\times 3$ array, where $M\in \mathbb {N}$ is the number of surface elements and, for each $i=1,\dots ,M$, the nodes of the *i*-th surface element (a triangle) are P(SurfElements(i,:),:);
- The optional output ElementsPlot is a polygonal mesh, represented as a list of objects of the class element2d, that approximates the surface $\Gamma _\text {cut}$ defined in (9). The surface mesh ElementsPlot can be used to plot numerical solutions inside of $\Omega$.

Similarly to the 2D case, we remark that the marching cubes algorithm does not guarantee mesh regularity [51]. For a workaround, we redirect the reader to the discussion at the end of Section 3.3. Finally, it must be noted that generate_mesh3d is guaranteed to generate star-shaped elements with star-shaped faces, which is essential for matrix assembly, see Section 4.3.

#### Remark 1

(Compatibility with third-party mesh generators) For triangulated surface meshes in 3D, the format (P, SurfElements) returned by the function generate_mesh3d is commonly used in finite element coding. For instance, the well-known Matlab package DistMesh [41] generates triangulated surface meshes in the same format. This implies that VEMcomp can solve surface PDEs using triangulated surface meshes generated with DistMesh, as we will show in the numerical example in Section 6.2.2. For bulk and bulk-surface meshes, the meshes generated by DistMesh or other packages would require a preliminary conversion step before being usable in VEMcomp.

## Computation of local and global matrices

In this Section we will address the computation of (i) local and (ii) global mass and stiffness matrices, specifically:

- We will show that the classes element2d and element3d introduced in Sections 3.3-3.4 will also allow to determine the local mass and stiffness matrices. To test the correctness of VEMcomp, we present for the first time the closed-form local VEM matrices for a square and a cubic element and compare the results with those obtained numerically by VEMcomp;
- We present the built-in functions assembly2d and assembly3d, which allow for the assembly of the global mass and stiffness matrices in 2D and 3D, respectively.

We remark that, following [30], the mass matrix is sufficient for the approximation of the nonlinearities in the time-dependent problems (2), (4) and (6).

### A worked example in 2D: the unit square

Here, we show the usage of element2d to compute the local matrices of the unit square and will compare the results with the local matrices in closed-form. In this regard, we remark that, while the closed-form stiffness matrix for the unit square was presented in [54], the closed-form mass matrix is a novel result. This result is further motivated by the marching squares mesh generation algorithm mentioned in Section 3.3. Consider the unit square $F = [0,1]^2$ contained in the *xy*-plane. Notice that vertex ordering affects the resulting matrices, so we order the vertexes as (0, 0, 0), (0, 1, 0), (1, 1, 0), and (1, 0, 0). With this choice, the closed-form local stiffness, mass and consistency matrices of *F* for the VEM of lowest order $k=1$ are as follows:

10 $$\begin{aligned} K = \frac{1}{4}\!\!\left[ \begin{array}{c c c c} \ \ 3 &  -1 &  -1 &  -1\\ -1 &  \ \ 3 &  -1 &  -1\\ -1 &  -1 &  \ \ 3 &  -1\\ -1 &  -1 &  - 1 &  \ \ 3\\ \end{array}\right] \!\!; \ M = \frac{1}{48}\!\!\left[ \begin{array}{c c c c} \ 17 &  -9 &  \ 13 &  -9\\ -9 &  \ 17 &  -9 &  \ 13\\ \ 13 &  -9 &  \ 17 &  -9\\ -9 &  \ 13 &  - 9 &  \ 17\\ \end{array}\right] \!\!; \ C = \frac{1}{48}\!\!\left[ \begin{array}{c c c c} \ 5 &  \ 3 &  \ 1 &  \ 3\\ \ 3 &  \ 5 &  \ 3 &  \ 1\\ \ 1 &  \ 3 &  \ 5 &  \ 3\\ \ 3 &  \ 1 &  \ 3 &  \ 5\\ \end{array}\right] \!\!. \end{aligned}$$

If we compute numerically such matrices using VEMcomp as shown in Appendix A, the elementwise errors are: 0 for the stiffness matrix *K*, 5.5511*e*-17 for the mass matrix *M*, and 2.7756*e*-17 for the consistency matrix *C*. The numerically computed matrices are thus exact up to machine precision.

### A worked example in 3D: the unit cube

Here we show the usage of element3d to compute the local matrices of the unit cube $E = [0,1]^3$ and will compare the results with the corresponding closed-form matrices. In this regard, we remark that the closed-form VEM local matrices for the unit cube, which we present here, are a novel result in the literature to the best of our knowledge. This result is further motivated by the marching cubes mesh generation algorithm mentioned in Section 3.4. Because vertex ordering is reflected in the resulting matrices, we order the vertexes as follows:

11 $$\begin{aligned} (0, 0,0)\ (0,0, 1)\ (0,1, 0)\ (0, 1,1)\ (1 ,0,0)\ (1 ,0,1)\ (1 ,1,0)\ (1 ,1,1). \end{aligned}$$

The local stiffness, consistency and mass matrices for the lowest order $k=1$ in closed-form are as follows. The interested reader is referred to Appendix A for the derivation.

12 $$\begin{aligned}&K\!\! =\!\! \frac{1}{16}\!\!\left[ \begin{array}{c c c c c c c c} \ \ 3 &  \ \ 1 &  \ \ 1 &  -1 &  \ \ 1 &  -1 &  -1 &  -3 \\ \ \ 1 &  \ \ 3 &  -1 &  \ \ 1 &  -1 &  \ \ 1 &  -3 &  -1 \\ \ \ 1 &  -1 &  \ \ 3 &  \ \ 1 &  -1 &  -3 &  \ \ 1 &  -1 \\ -1 &  \ \ 1 &  \ \ 1 &  \ \ 3 &  -3 &  -1 &  -1 &  \ \ 1 \\ \ \ 1 &  -1 &  -1 &  -3 &  \ \ 3 &  \ \ 1 &  \ \ 1 &  -1 \\ -1 &  \ \ 1 &  -3 &  -1 &  \ \ 1 &  \ \ 3 &  -1 &  \ \ 1 \\ -1 &  -3 &  \ \ 1 &  -1 &  \ \ 1 &  -1 &  \ \ 3 &  \ \ 1 \\ -3 &  -1 &  -1 &  \ \ 1 &  -1 &  \ \ 1 &  \ \ 1 &  \ \ 3 \end{array}\right] \!\!\! +\!\! \frac{\sqrt{3}}{4}\!\!\left[ \begin{array}{c c c c c c c c} \ \ 2 &  -1 &  -1 &  \ \ 0 &  -1 &  \ \ 0 &  \ \ 0 &  \ \ 1 \\ -1 &  \ \ 2 &  \ \ 0 &  -1 &  \ \ 0 &  -1 &  \ \ 1 &  \ \ 0 \\ -1 &  \ \ 0 &  \ \ 2 &  -1 &  \ \ 0 &  \ \ 1 &  -1 &  \ \ 0 \\ \ \ 0 &  -1 &  -1 &  \ \ 2 &  \ \ 1 &  \ \ 0 &  \ \ 0 &  -1 \\ -1 &  \ \ 0 &  \ \ 0 &  \ \ 1 &  \ \ 2 &  -1 &  -1 &  \ \ 0 \\ \ \ 0 &  -1 &  \ \ 1 &  \ \ 0 &  -1 &  \ \ 2 &  \ \ 0 &  -1 \\ \ \ 0 &  \ \ 1 &  -1 &  \ \ 0 &  -1 &  \ \ 0 &  \ \ 2 &  -1 \\ \ \ 1 &  \ \ 0 &  \ \ 0 &  -1 &  \ \ 0 &  -1 &  -1 &  \ \ 2 \\ \end{array}\right] \!\!; \ \end{aligned}$$

13 $$\begin{aligned}&C = \frac{1}{96}\left[ \begin{array}{c c c c c c c c} 3 \ \ &  2 \ \   &  2 \ \   &  1 \ \   &  2 \ \ &  1 \ \   &  1 \ \   &  0 \\ 2 \ \ &  3 \ \ &  1 \ \ &  2 \ \   &  1 \ \ &  2 \ \ &  0 \ \ &  1 \\ 2 \ \ &  1 \ \ &  3 \ \ &  2 \ \   &  1 \ \ &  0 \ \ &  2 \ \ &  1 \\ 1 \ \ &  2 \ \ &  2 \ \ &  3 \ \   &  0 \ \ &  1 \ \ &  1 \ \ &  2 \\ 2 \ \ &  1 \ \ &  1 \ \ &  0\ \ &  3 \ \ &  2 \ \ &  2 \ \   &  1 \\ 1 \ \ &  2 \ \ &  0 \ \ &  1 \ \   &  2 \ \ &  3 \ \ &  1 \ \   &  2 \\ 1 \ \ &  0 \ \ &  2 \ \ &  1 \ \   &  2 \ \ &  1 \ \ &  3 \ \ &  2 \\ 0 \ \ &  1 \ \ &  1 \ \   &  2 \ \   &  1 \ \ &  2 \ \ &  2 \ \ &  3 \end{array}\right] ;\end{aligned}$$

14 $$\begin{aligned}&M = \frac{1}{96}\left[ \begin{array}{c c c c c c c c} \ \ 51 &  -22 &  -22 &  1 &  -22 &  1 &  \ \ 1 &  \ \ 24 \\ -22 &  \ \ 51 &  1 &  -22 &  1 &  -22 &  \ \ 24 &  1 \\ -22 &  1 &  \ \ 51 &  -22 &  1 &  \ \ 24 &  -22 &  1 \\ 1 &  -22 &  -22 &  \ \ 51 &  \ \ 24 &  1 &  1 &  -22 \\ -22 &  1 &  1 &  \ \ 24 &  \ \ 51 &  -22 &  -22 &  1 \\ 1 &  -22 &  \ \ 24 &  1 &  -22 &  \ \ 51 &  1 &  -22 \\ 1 &  \ \ 24 &  -22 &  1 &  -22 &  1 &  \ \ 51 &  -22 \\ \ \ 24 &  1 &  1 &  -22 &  1 &  -22 &  -22 &  \ \ 51 \end{array}\right] . \end{aligned}$$

If we compute numerically such matrices using VEMcomp as shown in Appendix A, the elementwise errors are: 2.2204*e*-16 for the stiffness matrix *K*, and 1.5543*e*-15 for both the mass matrix *M* and the consistency matrix *C*. The numerically computed matrices are thus exact up to machine precision.

### Global matrix assembly

Once a mesh has been generated in the format returned by the functions generate_mesh2d or generate_mesh3d in Section 3, VEMcomp provides two functions for global matrix assembly in 2D and 3D. The 2D case is covered by the assembly2d function, whose syntax is as follows: 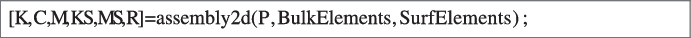 In the above, the inputs are as returned by the function generate_mesh2d in Section 3.3, while the outputs are defined as follows:

- K, C and M are the stiffness, consistency and mass matrices in the bulk, stored as sparse matrices;
- KS and MS are the stiffness and mass matrices on the surface, stored as sparse matrices. We observe that the surface here is a curve, so the “1D surface VEM” coincides with the 1D surface FEM, see [29]. This implies that the consistency matrix on the surface, say CS, coincides with the mass matrix on the surface MS [29], hence the lack of the additional output CS;
- R is the reduction matrix, see [29].

For full definitions of the above matrices, see [29]. Analogously, for matrix assembly in 3D, VEMcomp provides the function assembly3d, whose syntax is 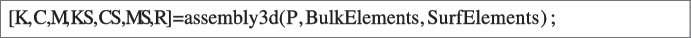 The inputs P, ElementsBulk, ElementsSurface are as returned by the function generate_mesh3d in Section 3.4. The outputs K,C,M,KS,CS,MS,R are defined as in the 2D case, with the addition of the consistency matrix CS for the surface, since in the 3D case it is no longer true that CS = MS, see [30]. It is worth noting that the PDEtool in Matlab has a similar syntax in the built-in function assempde for the FEM, see [55].

## Black-box solvers and post-processing

In this Section we will present VEMcomp’s functions for (i) solving the PDE models (1)-(6), (ii) plotting the numerical solutions and (iii) computing the numerical errors when a closed-form solution is known. The usage of all these functions will be demonstrated in several numerical examples in Section 6.

### Black-box solvers

VEMcomp provides black-box functions for the numerical approximation, via (BS)-FEM or (BS)-VEM, of the (BS)-PDE problems considered in Section 2. Here, we will state the syntax of such black-box solvers. Full examples will be shown in Section 6. VEMcomp solves the elliptic bulk-only problem (1) through the function 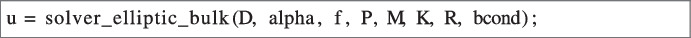 where, in input:

- D, alpha are two real numbers (double) containing the diffusion coefficient $d^\Omega > 0$ and the coefficient $\alpha \ge 0$ as in (1);
- f is an inline function representing the load term *f* in (1);
- P, M, K, R are as in the function assembly2d or assembly3d introduced in Section 4.3;
- bcond is a string indicating the kind of boundary conditions. The available options for bcond are ’dir’ and ’neu’, which correspond to homogeneous Dirichlet or Neumann boundary conditions, respectively.

In output, u is a size(P,1)$\times$1 array containing the nodal values of the numerical solution. The semilinear parabolic system (2) is solved through the function 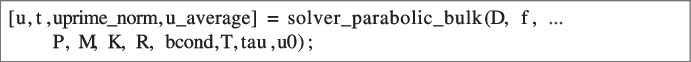 where, in input:

- D is a n$\times$1 array containing the diffusion coefficients $d_i^\Omega$, $i=1,\dots ,n$;
- f is a n$\times$1 cell array containing the kinetics $f_i$ as inline functions;
- P, M, K, R, bcond are as in the previous function solver_elliptic_bulk;
- T is the final time and tau is the timestep used in the IMEX Euler timestepping scheme [29];
- u0 is a size(P,1)$\times$n array where the *i*-th column contains the nodal values of the *i*-th component of the initial condition, for all $i=1,\dots ,n$;

In output:

- u is a size(P,1)$\times$1 array, where the *i*-th column contains the nodal values of the component $u_i$ of the numerical solution evaluated at the final time *T*, for all $i=1,\dots ,n$.
- the optional output t is a $1\times N_T$ array, where $N_T := \left\lceil \frac{T}{\tau }\right\rceil$, containing the time steps $t_k := k\tau$, $k=1,\dots ,N_T$.
- the optional output uprime_norm is also a $1\times N_T$ array containing the $L^2$ norm of the discrete time derivative of the first component of u evaluated at all time nodes:
  $$\begin{aligned} \mathtt {uprime\_norm}(k) \! = \! \frac{\Vert u_1^{(k)}-u_1^{(k-1)}\Vert _{L^2(\Omega _h)}}{\tau } \! := \! \frac{1}{\tau }\left( \int _{\Omega _h} \left( u_1^{(k)}-u_1^{(k-1)}\right) ^2\right) ^{\frac{1}{2}} \!\!\!\!, \ k=1,\dots , N_T. \end{aligned}$$
- the optional output u_average is a $1\times N_T$ array containing the spatial average of the first component of u evaluated at all time nodes:
  $$\begin{aligned} \mathtt {u\_average}(k) = <u_1^{(k)}> := \frac{1}{{{\,\textrm{measure}\,}}(\Omega _h)}\int _{\Omega _h} u_1^{(k)}, \ k=1,\dots , N_T. \end{aligned}$$

The optional outputs above are usually used in the context of RDSs to assess whether a stationary steady state (Turing pattern) has been reached, see for instance [56, 57]. With a similar and self-explanatory syntax, the surface and bulk-surface model problems (3)-(6) are solved through the functions 
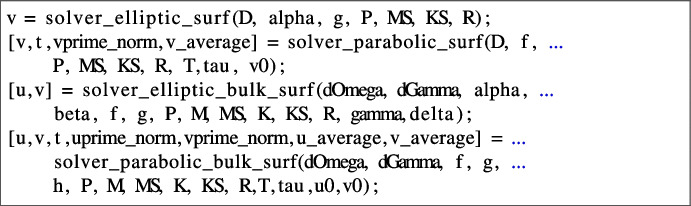
 All the mentioned black-box solvers rely on Matlab’s backslash \ direct solver, accelerated through the symamd reordering, for the linear systems appearing in the fully discrete problems.

### Solution plotters

We now present VEMcomp’s inhouse functions for plotting numerical solutions. In the 2D case, when $\Omega$ is a flat domain and $\Gamma = \partial \Omega$ is a curve, each bulk- and surface component of a numerical solution is plotted via the functions 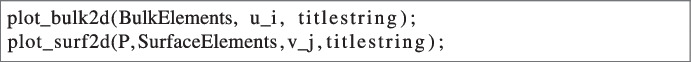 respectively. In the above, in input:

- P, BulkElements and SurfaceElements are outputs of the function generate_mesh2d in Section 3.3;
- u_i is a size(P,1) $\times$ 1 vector containing the nodal values of the bulk component $u_i$ of the numerical solution, and corresponds to the *i*-th column of the array u returned by any of the black-box solvers in Section 5.1;
- v_j is a size(R,2) $\times$ 1 vector containing the nodal values of the surface component $v_j$ of the numerical solution, and corresponds to the *j*-th column of the array v returned by any of the black-box solvers in Section 5.1;
- the optional input titlestring is the title of the plot in string format.

Similarly, for the 3D case, the plots are generated by the following functions: 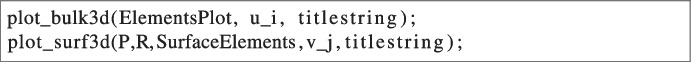 where, in input,

- P, ElementsPlot and SurfaceElements are generated by the function generate_mesh3d in Section 3.4;
- R is the reduction matrix generated by the function assembly3d in Section 4.3;
- u_i, v_j and titlestring are defined as in the 2D case above.

The presence of inhouse functions for solution plotting avoids the necessity to export the numerical solution to external plotters such as ParaView [58].

### Error evaluation

Being based on the Virtual Element Method, VEMcomp is backed up by extensive literature on stability and convergence. The interested reader is referred to the literature review in Section 2. When a closed-form solution is known, VEMcomp allows to compute the relative error of the numerical solution in (i) $L^2(\Omega )$ norm for bulk-only problems, (ii) $L^2(\Gamma )$ norm for surface-only problems and (iii) $L^2(\Omega ) \times L^2(\Gamma )$ norm for bulk-surface problems. It is worth recalling that the VEM basis functions are not known in closed form. For this reason it is not possible to evaluate the error of the numerical solution exactly. The VEM literature typically faces this issue by evaluating the error of the *piecewise polynomial projection of the numerical solution*, see for instance [15, Section 3.1.2], and VEMcomp follows this approach. Such error evaluation is performed by the function compute_error whose syntax is as follows 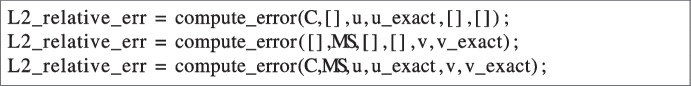 for bulk, surface and bulk-surface problems, respectively. In input:

- the bulk consistency matrix C and the surface mass matrix MS are outputs of assembly2d or assembly3d depending on the space dimension of the problem;
- u and/or v are outputs of any of the black-box solvers in Section 5.1;
- u_exact and/or v_exact are arrays of the same sizes of u and v, respectively, containing the nodal values of the exact solution.

In the next section, we present six numerical examples that illustrate the applicability, versatility and generality of VEMcomp.

## Numerical examples

In this Section, we present six numerical experiments carried out in VEMcomp to showcase the usage of our Matlab package.

### Bulk-only problems

This first set of examples showcases the application of VEMcomp to bulk-only PDE problems. In Example 6.1.1 we consider a Poisson problem in 2D on a circular domain with Neumann boundary conditions. In Example 6.1.2 we show the solution of a Poisson problem in 3D on the spherical domain with Dirichlet boundary conditions. The main aim here is demonstrate the generality of VEMcomp in dealing with different PDEs and different types of boundary conditions.

#### Poisson problem in 2D on a circular domain

**Fig. 5:**
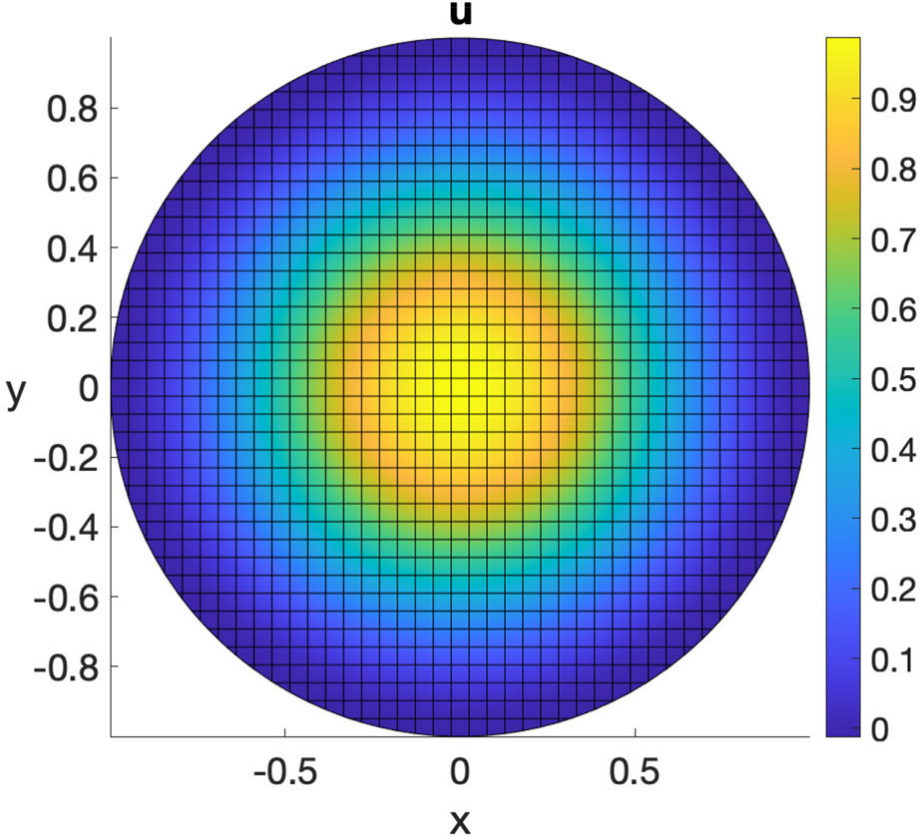
Numerical solution of the elliptic bulk problem (15) obtained in VEMcomp on a mesh with $N=1336$ nodes and meshsize $h=0.0725$

We consider the following Poisson problem on the unit circle $\Omega = \{(x,y)\in \mathbb {R}^2 \ | \ x^2 + y^2 \le 1\}$ with homogeneous Neumann boundary conditions:

15 $$\begin{aligned} {\left\{ \begin{array}{ll} -\Delta u + u = 8(1-2(x^2+y^2)) + (1-(x^2+y^2))^2, \qquad (x,y) \in \Omega ;\\ \nabla u \cdot \varvec{n}= 0, \qquad (x,y)\in \partial \Omega , \end{array}\right. } \end{aligned}$$

whose exact solution is given by $u(x,y) = (1-(x^2+y^2))^2$ for all $(x,y) \in \Omega$. We solve the considered problem, plot the numerical solution and evaluate the relative error in $L^2(\Omega )$ norm using the following VEMcomp code: 
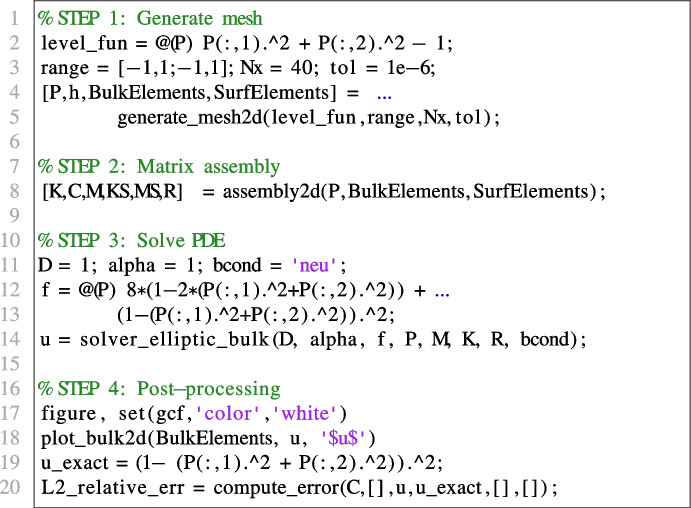
 The above code generates a mesh with $N = \texttt {size(P,1)} = 1336$ nodes and meshsize $h = 0.0725$. The obtained relative error in $L^2(\Omega )$ norm is 2.3734*e*-2. The numerical solution and the mesh are shown in Fig. 5.

#### Poisson equation in 3D on a spherical domain

**Fig. 6:**
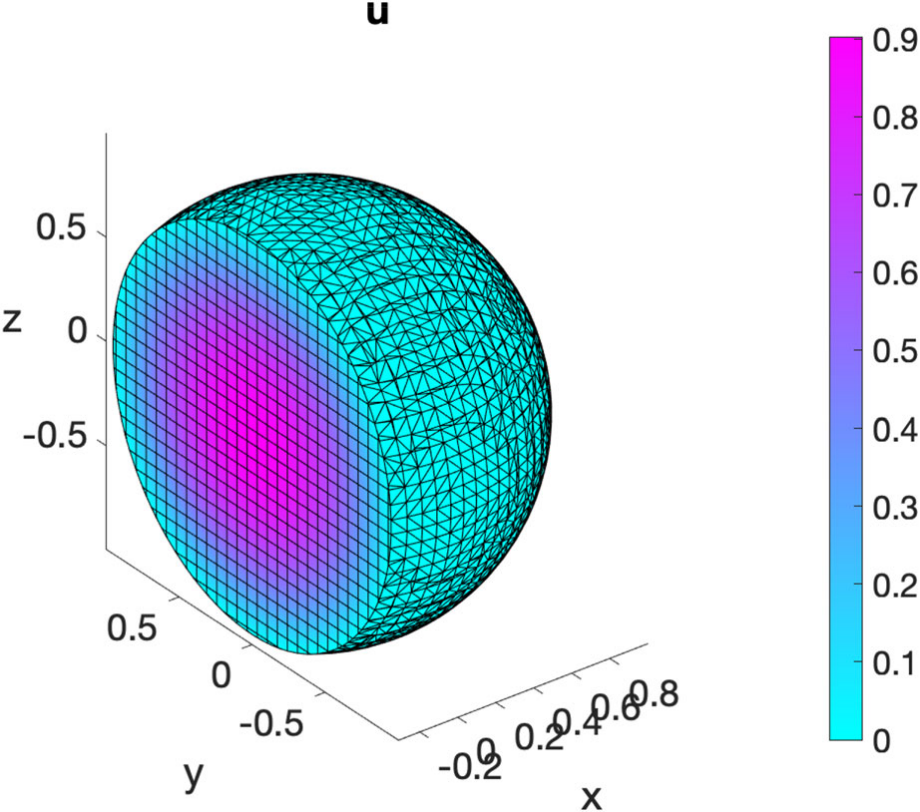
Numerical solution of the elliptic bulk problem (16) obtained in VEMcomp on a mesh with $N=16600$ nodes and meshsize $h=0.1195$. The colormap is different than in the other experiments, in order to better highlight the structure of the mesh

We now consider on the unit sphere $\Omega := \{(x,y,z) \in \mathbb {R}^3 \ | \ x^2 + y^2 + z^2 \le 1\}$ the following Poisson problem with homogeneous Dirichlet boundary conditions:

16 $$\begin{aligned} {\left\{ \begin{array}{ll} -\Delta u + u = 7-(x^2+y^2+z^2) \qquad (x,y,z)\in \Omega ;\\ u = 0 \qquad (x,y,z)\in \partial \Omega , \end{array}\right. } \end{aligned}$$

whose exact solution is given by $u(x,y,z) = 1-(x^2 + y^2 + z^2)$ for all $(x,y,z)\in \Omega$. We solve problem (16) in VEMcomp by running the following script code
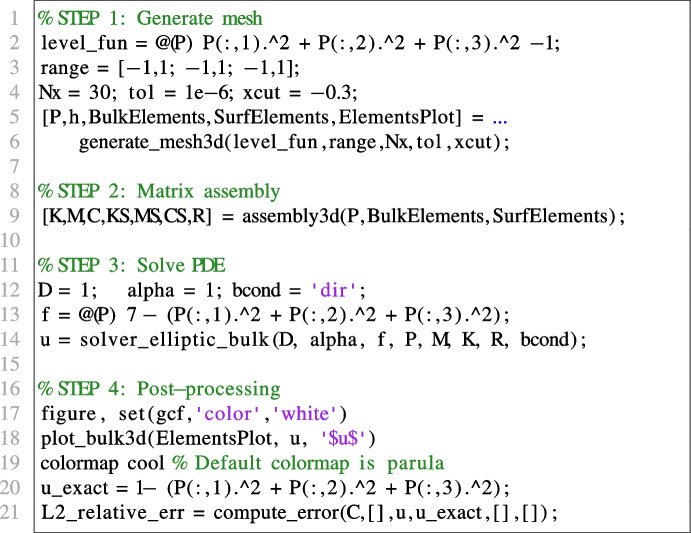
 The above code generates a mesh and computes its meshsize h, which is equal to 0.1195. By exploiting the definitions of the matrices produced by assembly3d, the number of nodes is given e.g. by size(P,1) and is equal to 16600. The relative error in $L^2(\Omega )$ norm is 1.7372e-4. The numerical solution and the mesh are shown in Fig. 6.

### Surface-only problems

This second set of examples showcases the application of VEMcomp to surface-only problems. In Example 6.2.1 we consider a linear parabolic problem on a spherical surface. In Example 6.2.2 we solve a reaction-diffusion system on an ellipsoidal surface and we demonstrate the compatibility of VEMcomp with the triangulated surfaces generated by DistMesh.

#### Linear parabolic problem

**Fig. 7:**
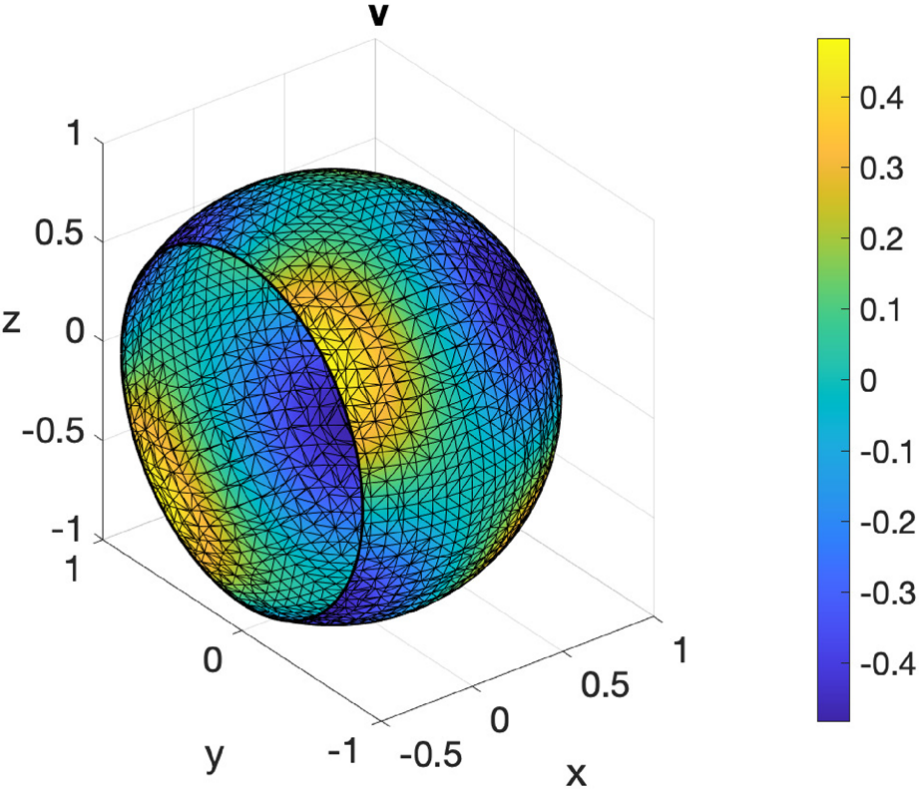
Numerical solution of the parabolic surface PDE (17) obtained in VEMcomp on a mesh with $N=3888$ nodes and meshsize $h=0.1195$

In this Section we show VEMcomp’s ability to solve surface PDEs. For illustrative purposes, we choose a linear parabolic problem on a spherical surface in 3D. We remark, however, that VEMcomp can solve surface PDE problems in 2D as well, i.e. when the spatial domain $\Gamma$ is a curve in $\mathbb {R}^2$. Let us then consider the following linear parabolic problem on the unit spherical surface $\Gamma$:

17 $$\begin{aligned} {\left\{ \begin{array}{ll} \dfrac{\partial u}{\partial t} - \Delta _\Gamma u = 13xyze^{t}, \qquad (x,y,z,t) \in \Gamma \times [0,T];\\ u(x,y,z,0) = xyz, \qquad (x,y,z) \in \Gamma , \end{array}\right. } \end{aligned}$$

whose exact solution is given by $u(x,y,z,t) = xyze^{t}$ for all $(x,y,z,t) \in \Gamma \times [0,T]$. The surface $\Gamma$ is approximated with the same surface mesh SurfElements generated -but not used- in the previous example in Section 6.1.2 with Nx = 30, while the bulk mesh BulkElements is now unused. By using the definition of the matrices produced by assembly3d, the number of nodes of the surface mesh is given e.g. by length(KS) or size(R,2) and is equal to 3888. Furthermore, as discussed in Section 6.1.2, the meshsize h is 0.1195. The final time is $T=1$ and the timestep is 1*e*-4. The code for mesh generation and matrix assembly is the same as in the previous experiment in Section 6.1.2. Therefore we report here only the code for the solver and plotter: 
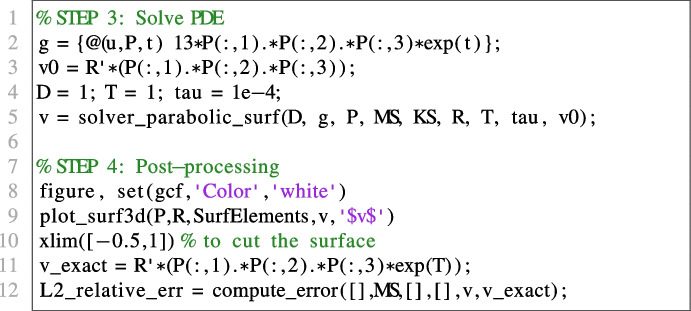
 The relative error, measured at the final time, is 6.5565*e*-3. The numerical solution and the surface mesh are shown in Fig. 7.

#### Surface RDS on the ellipsoid using DistMesh

To showcase the compatibility between DistMesh and VEMcomp in the case of surface PDEs, we solve the following surface reaction-diffusion system (RDS) with activator-depleted kinetics:

18 $$\begin{aligned} {\left\{ \begin{array}{ll} \dfrac{\partial v_1}{\partial t} - \Delta _\Gamma v_1 = \gamma _\Gamma g_1(v_1,v_2), & \text {in}\ \Gamma \times [0,T];\\ \dfrac{\partial v_2}{\partial t} - d^\Gamma _2 \Delta _\Gamma v_2 = \gamma _\Gamma g_2(v_1,v_2), &  \text {in} \ \Gamma \times [0,T], \end{array}\right. } \end{aligned}$$

where $g_1(v_1,v_2) = a-v_1+v_1^2v_2$, $g_2(v_1,v_2) = b - v_1^2v_2$, and *a*, *b* are positive reaction parameters. The model was considered in [59] and is an instance of the general surface parabolic problem (4). As shown in [59], the following is a spatially uniform steady state:

19 $$\begin{aligned} (v_1^*, v_2^*) := \left( a+b, \frac{b}{(a+b)^2}\right) . \end{aligned}$$

Suitable conditions on $a,b,d_2^\Gamma$ ensure that the steady state (19) is Turing unstable, i.e. in the presence of diffusion it destabilizes and for long time integration the solution tends towards a Turing pattern inhomogeneous in space. Following [59] we choose the following parameters:

20 $$\begin{aligned} a=0.1; \ b=0.9; \ d_2^\Gamma =10. \end{aligned}$$

**Fig. 8:**
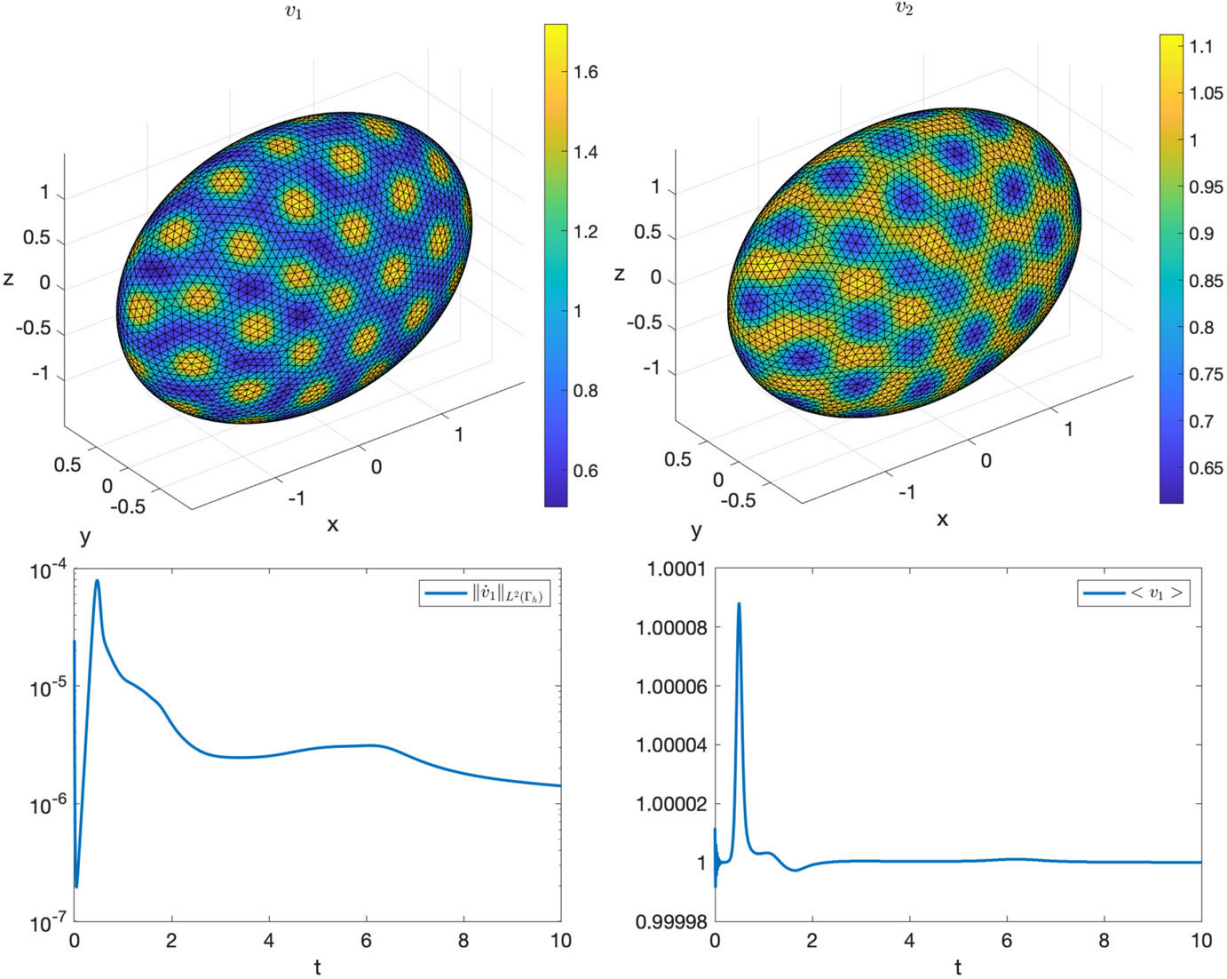
Numerical solution at the final time $T=10$ of the S-RDS (18) on the ellipsoid $\Gamma$ defined in (21), solved in VEMcomp on a mesh with 3990 nodes and meshsize $h \le 0.1$, whose exact value is not provided by DistMesh. Top row: component $v_1$ (left) and component $v_2$ (right). Bottom row: $L^2$ norm of time derivative of $v_1$ over time (left) and spatial average of $v_1$ over time (right)


Finally, for illustrative purposes, we choose $\gamma _\Gamma = 300$. For the spatial domain $\Gamma$, we consider the ellipsoid

21 $$\begin{aligned} \Gamma := \left\{ (x,y,z)\in \mathbb {R}^3 \ \Bigg | \ \frac{x^2}{4} + y^2 + \frac{z^2}{1.5^2} - 1 = 0\right\} , \end{aligned}$$

considered in the DistMesh manual [41]. To show pattern formation, the initial data for (18) are chosen as small spatially random perturbations of amplitude 1e-3 around the equilibrium (19). The final time is $T=10$ and the timestep is $\tau = 1e$-5. As expected, the solution at the final time, shown in Fig. 8, exhibits spatial patterns. Such solution is an asymptotic steady state since, as we can see in Fig. 8, at the final time, the $L^2$ norm of the discrete time derivative $\dot{v}_1$ is neglibible and the spatial average of $v_1$ has reached an asymptotic value. The code for generating the surface mesh with DistMesh, for solving the model and plotting the solution and the post-processing indicators in VEMcomp is as follows:

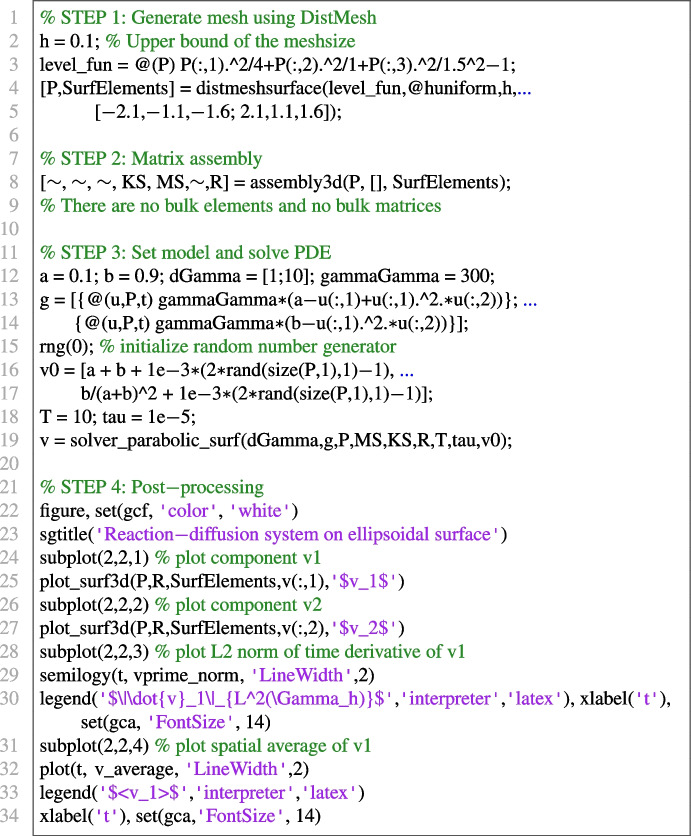

**Fig. 9:**
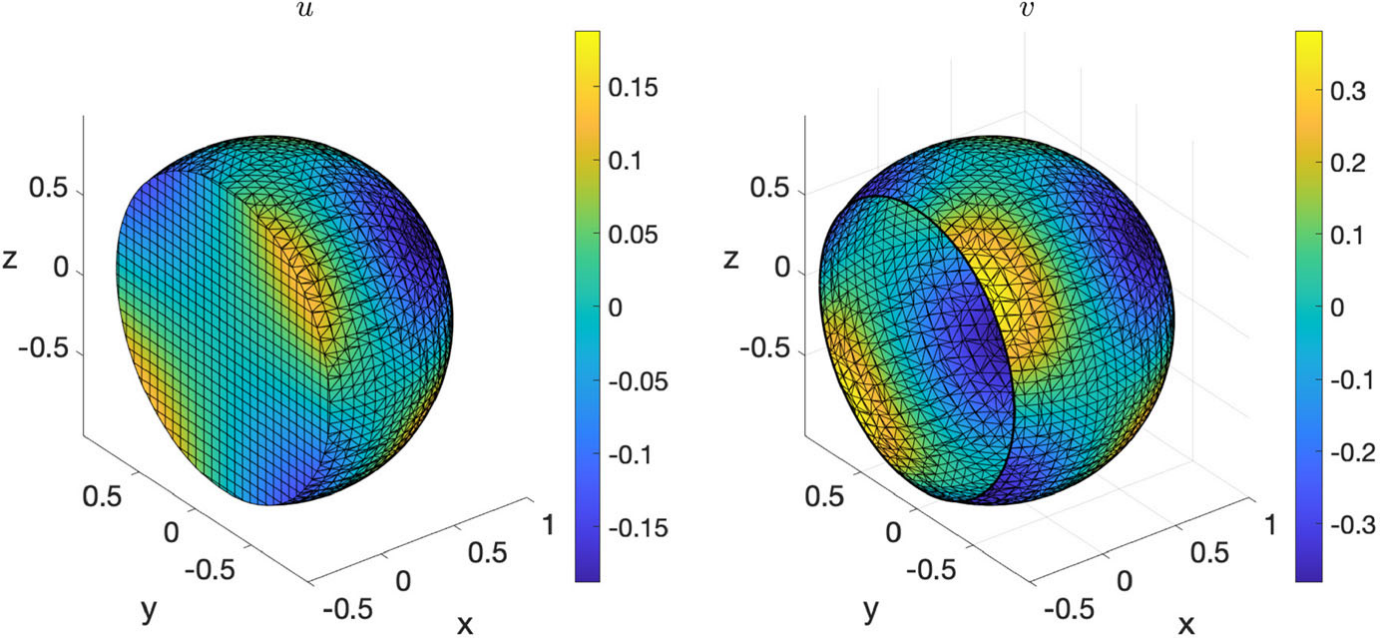
Elliptic bulk-surface problem (22) on the unit sphere $\Omega$ in 3D, solved in VEMcomp on a mesh with 16600 nodes and meshsize 0.1195. Left: bulk component *u*. Right: surface component *v*

In the above code, the meshsize, not provided exactly by DistMesh, is upper-bounded by h = 0.1. The number of nodes, given by size(P,1), is 3990.

### Numerical examples for bulk-surface problems in 3D

We now apply VEMcomp to two different BS-PDEs in 3D. In Example 6.3.1 we show a baseline problem given by a bulk-surface linear elliptic problem on the sphere. In Example 6.3.2, we use VEMcomp to solve the top-end PDE problem considered in this work: a bulk-surface reaction-diffusion system (BS-RDS) on the sphere. We remark that VEMcomp can solve bulk-surface problems in 2D as well.

#### Elliptic bulk-surface problem in 3D on the sphere

We numerically solve the following elliptic bulk-surface problem, from [60], on the unit sphere $\Omega$ in 3D:

22 $$\begin{aligned} {\left\{ \begin{array}{ll} -\Delta u + u = xyz, \qquad \text {in}\ \Omega ;\\ -\Delta _\Gamma v + v +\nabla u\cdot \varvec{n}= 29xyz, \qquad \text {on}\ \partial \Omega ;\\ \nabla u \cdot \varvec{n}= -u + 2v, \qquad \text {on}\ \partial \Omega , \end{array}\right. } \end{aligned}$$

whose exact solution is given by

$$\begin{aligned}&u(x,y,z) = xyz, \qquad (x,y,z) \in \Omega ;\\&v(x,y,z) = 2xyz, \qquad (x,y,z) \in \partial \Omega . \end{aligned}$$

We use the same mesh considered in Experiment 6.1.2. The obtained relative error in $L^2(\Omega ) \times L^2(\Gamma )$ norm is 6.9535*e*-3. The components of the numerical solution and the bulk-surface mesh are plotted in Fig. 9. Omitting the code for mesh generation and matrix assembly, which is the same as in the experiment in Section 6.1.2, the relevant piece of code for the dedicated solver and post-processing is:
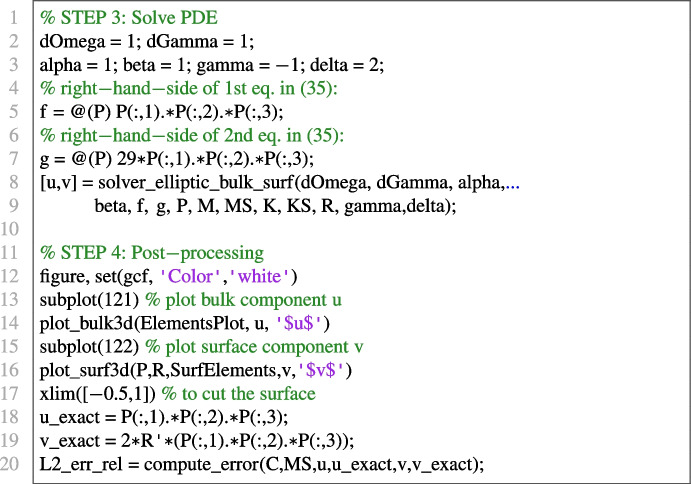


#### Bulk-surface reaction-diffusion system on the torus

In this final example we solve the following non-dimensionalised BS-RDS introduced in [61]:

23 $$\begin{aligned} {\left\{ \begin{array}{ll} \dfrac{\partial u_1}{\partial t} - \Delta u_1 = \gamma _\Omega f_1(u_1,u_2), & \text {in}\ \Omega \times [0,T];\\ \dfrac{\partial u_2}{\partial t} - d^\Omega _2 \Delta u_2 = \gamma _\Omega f_2(u_1,u_2), & \text {in} \ \Omega \times [0,T];\\ \dfrac{\partial v_1}{\partial t} - \Delta _\Gamma v_1 = \gamma _\Gamma (f_1(v_1,v_2) - h_1(u_1,u_2,v_1)), & \text {in}\ \Gamma \times [0,T];\\ \dfrac{\partial v_2}{\partial t} - d^\Gamma _2 \Delta _\Gamma v_2 = \gamma _\Gamma (f_2(v_1,v_2) - h_2(u_1,u_2,v_2)), &  \text {in} \ \Gamma \times [0,T];\\ \nabla u_1 \cdot \varvec{n}= \gamma _\Gamma h_1(u_1,u_2,v_1), &  \text {in} \ \Gamma \times [0,T];\\ d_\Omega \nabla u_2 \cdot \varvec{n}= \gamma _\Gamma h_2(u_1,u_2,v_2), & \text {in}\ \Gamma \times [0,T], \end{array}\right. } \end{aligned}$$

where $f_1(u_1,u_2) = a-u_1+u_1^2u_2$, $g(u_1,u_2) = b - u_1^2u_2$, $h_1(u_1,u_2,v_1) = \alpha _1 v_1 - \beta _1 u_1 -\kappa _1 u_1$, $h_2(u_1,u_2,v_2) = \alpha _2 v_2 - \beta _2 u_1 -\kappa _2 u_2$, and $a,b,\alpha _1,\alpha _2,\beta _1,\beta _2,\kappa _1,\kappa _2$ are positive reaction parameters. As shown in [61], the following is a spatially uniform steady state, under suitable conditions on the parameters $\alpha _1, \alpha _2,\beta _1,\beta _2,\kappa _1,\kappa _2$:

24 $$\begin{aligned} (u_1^*, u_2^*, v_1^*, v_2^*) := \left( a+b, \frac{b}{(a+b)^2}, a+b, \frac{b}{(a+b)^2}\right) . \end{aligned}$$

Further conditions on all the parameters except $\gamma _\Omega$ and $\gamma _\Gamma$ ensure that the steady state (24) is Turing unstable, i.e. in the presence of diffusion it destabilizes and for long time integration the solution tends towards a Turing pattern inhomogeneous in space. Following [61] we choose the following parameters:

25 $$\begin{aligned} {\begin{matrix} & a=0.1; \ b=0.9; \ \alpha _1=5/12; \ \alpha _2=5; \beta _1=5/12; \ \beta _2=0; \ \kappa _1=0;\ \kappa _2=5;\\ & d_2^\Omega =10; \ d_2^\Gamma =10. \end{matrix}} \end{aligned}$$

**Fig. 10:**
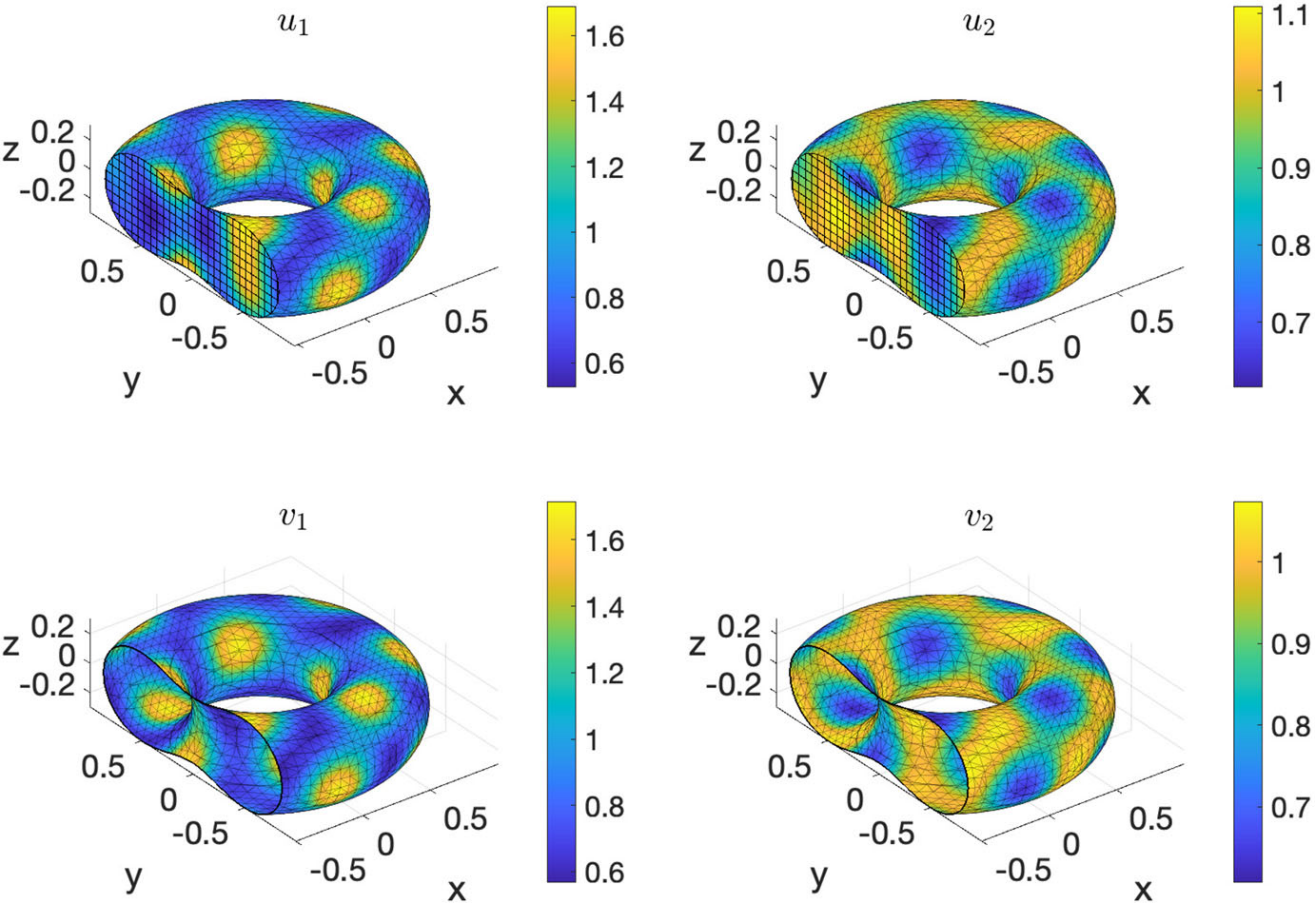
Numerical solution at the final time $T=5$ of the BS-RDS (23) on the torus $\Omega$ defined in (26), solved in VEMcomp on a mesh with 8144 nodes and meshsize 0.1074. Top row: bulk components $(u_1,u_2)$. Bottom row: surface components $(v_1,v_2)$

Finally, for illustrative purposes, we choose $\gamma _\Omega = \gamma _\Gamma = 300$. In [30], we had solved the BS-RDS (23) via BS-VEM on the unit sphere. Here, to showcase the generality of the VEMcomp library, we choose the torus

26 $$\begin{aligned} \Omega := \left\{ (x,y,z)\in \mathbb {R}^3 \ \Bigg | \ \left( \sqrt{x^2 + y^2} - \frac{7}{10}\right) ^2 + z^2 - \frac{9}{100} \le 0\right\} , \end{aligned}$$

as the bulk domain, and the toroidal surface $\Gamma := \partial \Omega$ as the surface domain. This specific toroidal surface $\Gamma$ was used in our previous work [13] as a benchmark domain for the Surface Virtual Element Method (SVEM) for surface PDEs. It can be shown that $\Omega$ is bounded as follows:

27 $$\begin{aligned} \Omega \subset [-1,1] \times [-1,1] \times [-0.31, 0.31], \end{aligned}$$

which is useful for mesh generation as discussed in Section 3.4. To show pattern formation, the initial data in (23) are chosen as small spatially random perturbations of amplitude 1e-3 around the equilibrium (24). Here we solve the model (23) by VEMcomp on a mesh generated by the generate_mesh3d function as shown below. The final time is $T=5$ and the timestep is $\tau = 1e$-5. As expected, the solution at the final time, shown in Fig. 10, is an asymptotic steady state that exhibits spatial patterns. We solve the considered problem by VEMcomp using the following code:
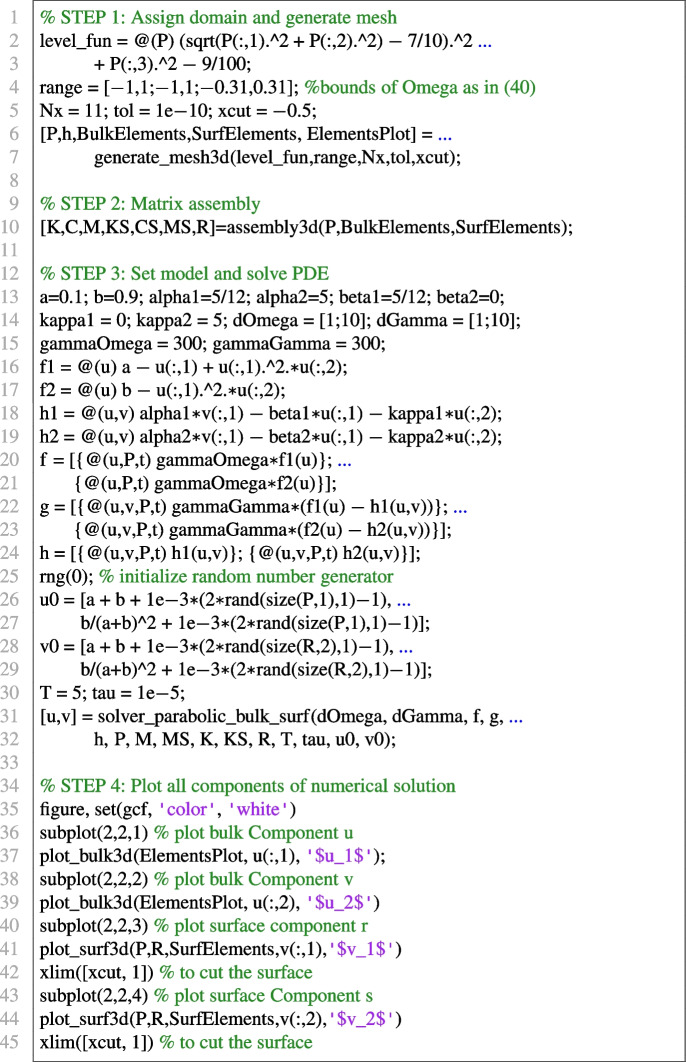
 Similarly with the previous experiments, in the above code, the meshsize h is 0.1074, the overall number of nodes, given by size(P,1), is 8144, while the number of boundary nodes, given by length(KS) or size(R,2), is 3008.

## Conclusions

We have introduced VEMcomp, a user-friendly MATLAB library for (i) polytopal bulk-surface mesh generation in 2D and 3D, (ii) matrix assembly for the lowest-order FEM and VEM, (iii) solving bulk, surface, and bulk-surface, elliptic and parabolic, linear and semilinear PDEs or systems of PDEs and (iv) post-processing the numerical solution, in terms of plotting and error evaluation. The present VEMcomp package is intended as a proof-of-concept tool, with the main goal of being user-friendly and self-explicative to solve a large selection of PDE models 2D and 3D. For this reason, VEMcomp has room for improvement in terms of computational performances. To this end, some main challenges are (i) devising mesh generation strategies that are cheaper and guarantee mesh regularity, (ii) devising cheaper quadrature formulas in 2D and 3D, (iii) including higher order VEM, (iv) solving PDEs on evolving domains and surfaces, (v) devising higher order schemes for time integration and finally (vi) adopting fast iterative solvers (e.g. conjugate gradient type) for the linear systems involved in the fully discrete problems.

## Data Availability

The VEMcomp package is available in MF’s GitHub repository under the GPLv2 license. The Matlab scripts used in Section 6 and in the Appendix A are also incorporated in the article.

## Appendix A: Local matrices in closed form

As shown in [54, Sections 3.2 and 5.1], the computation of the local VEM matrices relies on three fundamental matrices $B \in \mathbb {R}^{(d+1)\times \texttt {NVert}}$, $D \in \mathbb {R}^{\texttt {NVert} \times (d+1)}$, $H \in \mathbb {R}^{(d+1)\times (d+1)}$ (where $d\in \{2,3\}$ is the dimension of the problem and NVert is the number of vertexes of the given element) which are uniquely determined by the vertexes and whose lengthy definitions we do not report here. With the matrices *B*, *D* in hand, the following matrices can be obtained as shown in [54, Sections 3.2 and 3.3]:

- $$G := BD \in \mathbb {R}^{(d+1)\times (d+1)}$$;
- $$\widetilde{G} := \left[ \begin{array}{ccc} 0 &  0\\ 0 &  I_d \end{array}\right] G \in \mathbb {R}^{(d+1)\times (d+1)}$$, where $I_d$ is the $d\times d$ identity matrix;
- $$\Pi ^\nabla _* := G^{-1}B \in \mathbb {R}^{(d+1)\times \texttt {NVert}}$$;
- $$\Pi ^\nabla := D\Pi ^\nabla _* \in \mathbb {R}^{\texttt {NVert}\times \texttt {NVert}}$$.

Finally, as shown in [54, Sections 3.3 and 6.2] the local stiffness, consistency and mass matrices are given by

A1 $$\begin{aligned}&K = (\Pi ^\nabla _*)^T \widetilde{G} \Pi ^\nabla _* + (I-\Pi ^\nabla )^T(I-\Pi ^\nabla );\end{aligned}$$

A2 $$\begin{aligned}&C = (\Pi ^\nabla _*)^T H \Pi ^\nabla _*;\end{aligned}$$

A3 $$\begin{aligned}&M = C + \text {Area(F)}(I-\Pi ^\nabla )^T(I-\Pi ^\nabla ). \end{aligned}$$

For simplicity, in (A1)-(A3), the simplest form of VEM stabilization was used, the so-called *dofi-dofi* stabilization, see [62] for a review on VEM stabilization techniques.

### A.1 The unit square in 2D

It is possible to show that

A4 $$\begin{aligned}&B \! = \! \frac{1}{4}\left[ \begin{array}{c c c c} \ \ 1 &  \ \ 1 &  \ \ 1 &  \ \ 1\\ -\sqrt{2} &  \ \ \sqrt{2} &  \ \ \sqrt{2} &  -\sqrt{2}\\ -\sqrt{2} &  -\sqrt{2} &  \ \ \sqrt{2} &  \ \ \sqrt{2} \end{array}\right] \!\!; \ D = \frac{1}{4}\left[ \begin{array}{c c c} 4 &  -\sqrt{2} &  -\sqrt{2}\\ 4 &  \ \ \sqrt{2} &  -\sqrt{2}\\ 4 &  \ \ \sqrt{2} &  \ \ \sqrt{2}\\ 4 &  -\sqrt{2} &  \ \ \sqrt{2}\\ \end{array}\right] \!\!; \ H = \frac{1}{24}\left[ \begin{array}{c c c} 24 &  0 &  0\\ 0 &  1 &  0\\ 0 &  0 &  1\\ \end{array}\right] \!\!. \end{aligned}$$

In particular, the matrices *B*, *D* in (A4) were already computed in [54]. It follows that

A5 $$\begin{aligned} G = \frac{1}{2}&\left[ \begin{array}{c c c} 2 &  0 &  0\\ 0 &  1 &  0\\ 0 &  0 &  1\\ \end{array}\right] ; \qquad \widetilde{G} = \frac{1}{2}\left[ \begin{array}{c c c} 0 &  0 &  0\\ 0 &  1 &  0\\ 0 &  0 &  1\\ \end{array}\right] ;\end{aligned}$$

A6 $$\begin{aligned} \Pi ^\nabla _* = \frac{1}{4}&\left[ \begin{array}{c c c c} \ \ 1 &  \ \ 1 &  \ \ 1 &  \ \ 1\\ -2\sqrt{2} &  \ \ 2\sqrt{2} &  \ \ 2\sqrt{2} &  -2\sqrt{2}\\ -2\sqrt{2} &  -2\sqrt{2} &  \ \ 2\sqrt{2} &  \ \ 2\sqrt{2} \end{array}\right] ; \quad \Pi ^\nabla = \frac{1}{4}\left[ \begin{array}{c c c c} \ \ 3 &  \ \ 1 &  -1 &  \ \ 1\\ \ \ 1 &  \ \ 3 &  \ \ 1 &  -1\\ -1 &  \ \ 1 &  \ \ 3 &  \ \ 1\\ \ \ 1 &  -1 &  \ \ 1 &  \ \ 3\\ \end{array}\right] . \end{aligned}$$

The final result (10) then follows. To test the correctness of the numerically computed local matrices for the unit square, we use the following script, whose output is commented at the end of Section 4.1. In the above script, in the constructor of element_2d, we have set the property is_square to false. In this way, VEMcomp does not know that F is a square and therefore actually computes the local matrices numerically.

### A.2 The unit cube in 3D

Using lengthy but simple computations it is possible to show that

A7 $$\begin{aligned} B = \frac{1}{8\sqrt{3}}&\left[ \begin{array}{c c c c c c c c} \sqrt{3} &  \sqrt{3} &  \sqrt{3} &  \sqrt{3} &  \sqrt{3} &  \sqrt{3} &  \sqrt{3} &  \sqrt{3}\\ -2 &  -2 &  -2 &  -2 &  \ \ 2 &  \ \ 2 &  \ \ 2 &  \ \ 2\\ -2 &  -2 &  \ \ 2 &  \ \ 2 &  -2 &  -2 &  \ \ 2 &  \ \ 2\\ -2 &  \ \ 2 &  -2 &  \ \ 2 &  -2 &  \ \ 2 &  -2 &  \ \ 2\\ \end{array}\right] ;\end{aligned}$$

A8 $$\begin{aligned} D = \frac{1}{2\sqrt{3}}&\left[ \begin{array}{c c c c} 2\sqrt{3} &  -1 &  -1 &  -1\\ 2\sqrt{3} &  -1 &  -1 &  \ \ 1\\ 2\sqrt{3} &  -1 &  \ \ 1 &  -1\\ 2\sqrt{3} &  -1 &  \ \ 1 &  \ \ 1\\ 2\sqrt{3} &  \ \ 1 &  -1 &  -1\\ 2\sqrt{3} &  \ \ 1 &  -1 &  \ \ 1\\ 2\sqrt{3} &  \ \ 1 &  \ \ 1 &  -1\\ 2\sqrt{3} &  \ \ 1 &  \ \ 1 &  \ \ 1 \end{array}\right] ; \qquad H = \frac{1}{36}\left[ \begin{array}{c c c c} 36 &  0 &  0 &  0\\ 0 &  1 &  0 &  0\\ 0 &  0 &  1 &  0\\ 0 &  0 &  0 &  1 \end{array}\right] . \end{aligned}$$

It then follows that

A9 $$\begin{aligned} G = \frac{1}{3}&\left[ \begin{array}{c c c c} 3 &  0 &  0 &  1\\ 0 &  1 &  0 &  0\\ 0 &  0 &  1 &  0\\ 0 &  0 &  0 &  1 \end{array}\right] , \qquad \widetilde{G} = \frac{1}{3}\left[ \begin{array}{c c c c} 0 &  0 &  0 &  1\\ 0 &  1 &  0 &  0\\ 0 &  0 &  1 &  0\\ 0 &  0 &  0 &  1 \end{array}\right] ;\end{aligned}$$

A10 $$\begin{aligned} \Pi ^\nabla _* =\frac{1}{8}&\left[ \begin{array}{c c c c c c c c} 1 &  1 &  1 &  1 &  1 &  1 &  1 &  1\\ -2\sqrt{3} &  -2\sqrt{3} &  -2\sqrt{3} &  -2\sqrt{3} &  \ \ 2\sqrt{3} &  \ \ 2\sqrt{3} &  \ \ 2\sqrt{3} &  \ \ 2\sqrt{3}\\ -2\sqrt{3} &  -2\sqrt{3} &  \ \ 2\sqrt{3} &  \ \ 2\sqrt{3} &  -2\sqrt{3} &  -2\sqrt{3} &  \ \ 2\sqrt{3} &  \ \ 2\sqrt{3}\\ -2\sqrt{3} &  \ \ 2\sqrt{3} &  -2\sqrt{3} &  \ \ 2\sqrt{3} &  -2\sqrt{3} &  \ \ 2\sqrt{3} &  -2\sqrt{3} &  \ \ 2\sqrt{3}\\ \end{array}\right] ;\end{aligned}$$

A11 $$\begin{aligned} \Pi ^\nabla = \frac{1}{4}&\left[ \begin{array}{c c c c c c c c} 2 &  1 &  1 &  0 &  1 &  0 &  0 &  -1\\ 1 &  2 &  0 &  1 &  0 &  1 &  -1 &  0\\ 1 &  0 &  2 &  1 &  0 &  -1 &  1 &  0\\ 0 &  1 &  1 &  2 &  -1 &  0 &  0 &  1\\ 1 &  0 &  0 &  -1 &  2 &  1 &  1 &  0\\ 0 &  1 &  -1 &  0 &  1 &  2 &  0 &  1\\ 0 &  -1 &  1 &  0 &  1 &  0 &  2 &  1\\ -1 &  0 &  0 &  1 &  0 &  1 &  1 &  2 \end{array}\right] . \end{aligned}$$

The final result in equation (12) then follows. To test the correctness of the numerically computed local matrices for the unit cube, we use the following script, whose output is commented at the end of Section 4.2.
